# The Russian Aphasia Test: The first comprehensive, quantitative, standardized, and computerized aphasia language battery in Russian

**DOI:** 10.1371/journal.pone.0258946

**Published:** 2021-11-18

**Authors:** Maria V. Ivanova, Yulia S. Akinina, Olga A. Soloukhina, Ekaterina V. Iskra, Olga V. Buivolova, Anna V. Chrabaszcz, Ekaterina A. Stupina, Maria V. Khudyakova, Tatiana V. Akhutina, Olga Dragoy

**Affiliations:** 1 University of California, Berkeley, CA, United States of America; 2 HSE University, Moscow, Russian Federation; 3 University of Groningen, Groningen, The Netherlands; 4 Center for Speech Pathology and Neurorehabilitation, Moscow, Russian Federation; 5 Federal Center of Brain Research and Neurotechnologies, Moscow, Russian Federation; 6 University of Pittsburgh, Pittsburgh, PA, United States of America; 7 Lomonosov Moscow State University, Moscow, Russian Federation; 8 Institute of Linguistics, Russian Academy of Sciences, Moscow, Russian Federation; The University of Hong Kong, HONG KONG

## Abstract

The lack of standardized language assessment tools in Russian impedes clinical work, evidence-based practice, and research in Russian-speaking clinical populations. To address this gap in assessment of neurogenic language disorders, we developed and standardized a new comprehensive assessment instrument–the Russian Aphasia Test (RAT). The principal novelty of the RAT is that each subtest corresponds to a specific level of linguistic processing (phonological, lexical-semantic, syntactic, and discourse) in different domains: auditory comprehension, repetition, and oral production. In designing the test, we took into consideration various (psycho)linguistic factors known to influence language performance, as well as specific properties of Russian. The current paper describes the development of the RAT and reports its psychometric properties. A tablet-based version of the RAT was administered to 85 patients with different types and severity of aphasia and to 106 age-matched neurologically healthy controls. We established cutoff values for each subtest indicating deficit in a given task and cutoff values for aphasia based on the Receiver Operating Characteristic curve analysis of the composite score. The RAT showed very high sensitivity (> .93) and specificity (> .96), substantiating its validity for determining presence of aphasia. The test’s high construct validity was evidenced by strong correlations between subtests measuring similar linguistic processes. The concurrent validity of the test was also strong as demonstrated by a high correlation with an existing aphasia battery. Overall high internal, inter-rater, and test-retest reliability were obtained. The RAT is the first comprehensive aphasia language battery in Russian with properly established psychometric properties. It is sensitive to a wide range of language deficits in aphasia and can reliably characterize individual profiles of language impairments. Notably, the RAT is the first comprehensive aphasia test in any language to be fully automatized for administration on a tablet, maximizing further standardization of presentation and scoring procedures.

## Introduction

The Russian language is spoken by about 260 million people worldwide, making it the 8^th^ commonly spoken language in the world [1]. Still, there is a dramatic dearth of standardized tests for assessment of language disorders in Russian speakers [2]. Historically, a qualitative approach to clinical assessment grounded in Luria’s neuropsychological theory has dominated the clinical field in Russia [3, 4]. Patients are assessed with custom neuropsychological probes, targeting various cognitive and language domains; based on the pattern of performance, a conclusion regarding their language status is made by a clinician, which cannot be entirely objective by definition. While this approach is highly valuable for understanding the mechanisms of cognitive impairments and their neural substrate on an individual basis, it is not readily quantifiable or easily generalizable, and is highly dependent on the expertise of the clinician doing the assessment. This, overall, impedes evidence-based practice. Currently, the landscape for language assessment in Russian looks barren, and there is significant clinical and research need for standardized tools.

Up to now, the Assessment of Speech in Aphasia (ASA, in Russian *Metodika otsenki rechi pri afazii*; [5]) has been the most commonly used quantitative battery in Russian for assessment of language deficits in aphasia, although it remains largely unfamiliar to the global research community. The ASA includes ratings of conversational speech and a set of production and comprehension subtests at the word and sentence levels (see [6] for a detailed description of the subtests in English). State-of-the-art at the time of its creation, it incorporated comprehension and production of both nouns and verbs and tested syntactic constructions of various complexity. Also, the test was standardized on an impressively large sample (*N* = 234) of people with aphasia (PWA). However, it falls short of contemporary psychometric standards in several important aspects. First of all, the test lacks normative data on performance by individuals without aphasia. In the original publication [5], it is simply acknowledged that 30 healthy controls were tested to ensure that all items could be completed by individuals without brain injuries, but no actual normative data were provided. Subsequently, it was implicitly assumed that individuals without a language disorder should perform perfectly on the test, which is not a realistic premise. In practice, such expectation makes the clinicians determine the aphasia cutoff score subjectively on an individual basis. Secondly, the analyses lack statistical rigor, with the main focus being on exploring patterns of raw subtest scores in different aphasia subtypes. No transformations of raw scores are provided, making comparison between subjects and subtests challenging. Thirdly, formal reliability metrics of the test were not established, which is especially problematic for the subjective rating scale of the discourse subtests. Further, the ASA lacks subtests targeting several important language levels and domains (e.g., repetition and phonological processing are not assessed, comprehension is not tested at the discourse level). Finally, it has outdated visual stimuli (that some of the younger patients today have a hard time recognizing), and the word frequency counts that guided verbal stimuli selection are obsolete.

Another clinically adopted Russian battery is that of Vasserman and colleagues [7]. The speech and language subtests are a part of a comprehensive neuropsychological examination and were devised to provide a diagnosis of language deficits according to Luria’s aphasia classification [3, 4]. Unlike the ASA, Vasserman and colleagues [7] presented normative data of neurologically healthy individuals (*N* = 147) in three age groups; however, data on performance of PWA were not reported. Theoretically grounded in Luria’s approach to neuropsychological diagnostics, it is a useful guide for practicing clinicians who work within this framework. However, the method of Vasserman and colleagues [7] has several important shortcomings that limit its clinical applications and prevent its use for research purposes. Aspects of speech and language, such as spontaneous speech, naming, repetition, fluency etc., are assessed based on a custom four-point scale that blends qualitative and quantitative metrics. The items in the tasks are few in number, and psycholinguistic variables of the materials are not controlled. With respect to psychometric properties, only test-retest reliability in healthy controls is explicitly reported. To sum up, while the ASA [5] and the method of Vasserman and colleagues [7] continue to be used widely for quantification of language deficits in Russian-speaking PWA, they do not conform to contemporary standards for comprehensive standardized assessment of language.

To explore a possible alternative, Ivanova and Hallowell [8] validated a short version of the previously translated into Russian Bilingual Aphasia Test in PWA [9], but concluded that the translated version of the test battery has problematic psychometric properties. Since the test was never normed on a sample of healthy age-matched controls, criterion validity along with specificity and sensitivity could not be established. Additionally, some of the items were originally incorrectly translated into Russian, and some of the visual stimuli were unrecognizable. This work alerts of the pitfalls of using tests directly translated from one language into another bypassing the necessary stages of test development, adaptation, norming and standardization.

In addition to the aforementioned comprehensive test batteries, several other tests have been adapted to Russian recently that serve screening purposes [10, 11] or target a specific language component [8, 12]. For assessment of language deficit in the acute post-stroke period, the Aphasia Rapid Test [13] has been adapted to Russian and standardized on a cohort of PWA and healthy individuals [10]. This test can be administered in 3–5 minutes and demonstrates high sensitivity in identifying speech/language disorders, although without further differentiation between aphasia and motor speech deficits. For the latter purpose, the Aphasia Bedside Check (ABC) [14] was adapted to Russian. This screening tool evaluates language comprehension and production along with verbal fluency, repetition, articulation, and reading aloud. To investigate whether PWA in acute stage have phonological, semantic or syntactic deficits, the Russian translation of the ScreeLing test [15] is used. The norms for the tablet administration of the Russian version of the shortened Token Test [16] are also currently being analyzed [11]. In the test, tokens of various shape and color are presented to participants, who have to perform different actions with them (touch, rearrange, etc.) following oral instructions. In this manner, the test establishes presence of aphasia and its severity through assessment of auditory comprehension. The standardization of the Russian versions of the ABC, the ScreeLing and the shortened Token Test are currently underway; however, their use will be limited to screening purposes.

With regards to tests targeting specific language domains, Hallowell and Ivanova [17] normed the Russian multiple-choice version of the Revised Token Test [18] in both healthy controls and PWA. The test specifically assesses syntactic aspects of auditory comprehension. It requires matching of sentences of increasing complexity that describe the spatial location of tokens to pictures. Finally, the Verb and Sentence Test (VAST) [19–21] has been adapted to Russian [12]. It has several subtests that assess production and comprehension of isolated verbs and sentences. The VAST provides a profile of language impairment with the focus on verbs and grammar; however, no norms are currently available for the Russian version.

All of these tests are either brief and coarse screening batteries meant for quick bedside assessment or target a specific linguistic domain. Thus, they cannot substitute a detailed language evaluation or provide a comprehensive picture of the patient’s linguistic strengths and weaknesses across language domains on the same scale. The few currently existing quantitative comprehensive language tests in Russian [5, 7, 9] have not been normed properly, lack subtests targeting several important domains and linguistic levels, and have outdated stimuli. This lack of proper diagnostic tools makes it challenging to reliably evaluate and quantify the severity of linguistic deficits in individuals with various aphasia types, which, in turn, precludes systematic approaches to treatment in clinical practice and conduction of quantitative research. A comprehensive aphasia battery developed specifically for the Russian language is much needed. To address this gap, we developed and standardized a new comprehensive aphasia test–the Russian Aphasia Test (RAT).

In development of the RAT, we aimed to integrate the most current neurolinguistic and psychometric practices. The principal novelty of the RAT is that each subtest corresponds to a specific level of language processing (phonological, lexical-semantic, syntactic, discourse) within the following domains: auditory comprehension, repetition, and oral production (reading and writing domains are not currently included in the test). Similar to a recently developed Quick Aphasia Battery (QAB) [22], we designed the RAT such that instead of specifying aphasia types, a multidimensional characterization of impaired and spared aspects of language functioning could be made. The selection of specific tasks for each processing level was motivated by the structure and materials of contemporary well-established standardized aphasia tests in other languages (Boston Diagnostic Aphasia Examination [23], Comprehensive Aphasia Test [24], Northwestern Assessment of Verbs and Sentences [25], Northwestern Naming Battery [26], Psycholinguistic Assessments of Language Processing in Aphasia [27], Verb and Sentence Test [19, 20], Western Aphasia Battery-Revised [28]; see also [29]). Additionally, we took into consideration various (psycho)linguistic factors known to influence performance, as well as the specific structural and phonetic properties of the Russian language. We aimed to develop a test that would reliably identify impaired language processing and also provide a detailed evaluation of linguistic strengths and weaknesses in a clinically feasible time. We selected items ranging in difficulty, so that we could capture the full spectrum of aphasic language disorders and would be able to specify profile of impairment in individuals with different aphasia syndromes. Additionally, the RAT, to the best of our knowledge, is the first comprehensive aphasia test that is fully automatized for presentation and response recording on a tablet, further optimizing and standardizing administration and scoring procedures and simplifying data collection. Ultimately, the RAT will have multi-faceted applications in the clinical and research settings. The test is intended as a standardized assessment instrument to: (a) identify a language impairment (aphasia) in individuals with brain damage relative to healthy controls; (b) quantify aphasia severity; (c) provide a comprehensive multidimensional characterization of linguistic strengths and weakness across multiple language domains; and (d) detect change in language abilities over time.

The goals of the current paper are to provide a detailed background on the construction of the RAT, describe its components, explain scoring procedures, and establish its psychometric properties. With respect to the last goal, we aimed to evaluate diagnostic properties of the RAT in terms of differentiating between PWA and healthy controls, its construct and concurrent validity, and inter-rater and test-retest reliability.

## Materials and methods

### Participants

#### Neurologically healthy individuals (NHI) group

One hundred and six native speakers of Russian without history of neurological or psychiatric disorders or substance abuse participated in the study (see Table 1 for participants’ demographic characteristics). The level of formal education varied from secondary school (typically 10 years in Russia) to a university degree (typically 15 years), with the majority of the participants (80.2%) having some form of higher education degree (12–15 years). Most participants were right-handers (*N* = 101); three were left-handers and two were retrained left-handers. All participants reported normal or corrected-to-normal vision and hearing acuity.

**Table 1:** Participants’ demographic characteristics.

Group		NHI	Main PWA	Inter-rater PWA	Test-retest PWA– 1	Test-retest PWA– 2
*N*		106	85	20	20	20
**Gender**	***F/M***	77 / 29	26 / 59	7 / 13	8 / 12	11 / 9
**Age (years)**	***M (SD)***	49.9 (18.4)	57.6 (12.1)	57.1 (11.5)	54.9 (10.3)	58.5 (12.9)
	***Range***	19–86	25–80	32–70	34–69	39–82
**Time post-onset (months)**	***M (SD)***	NA	34.7 (45.2)	55.3 (71)	53.1 (37.1)	38.2 (35.7)
	***Range***	NA	1–249	2–249	12–162	8–133

*Note*. NHI–neurologically healthy individuals; PWA–people with aphasia. Inter-rater PWA group included randomly selected participants from the Main PWA group for evaluation of the test’s inter-rater reliability. Test-retest PWA—1 group included additionally recruited participants for evaluation of test-retest reliability; they completed all subtests except discourse comprehension, sentence production, and discourse production. Test-retest PWA group—2 included additionally recruited participants who performed discourse comprehension, sentence production, and discourse production subtests.

#### Age stratification

Initially, the data were collected to evenly represent three age groups: 18–39 years old (group 1, *N* = 33), 40–59 years old (group 2, *N* = 36), and 60+ years old (group 3, *N* = 37). After the data were collected, we tested for significant differences between the three age groups of the NHI cohort in each subtest (Kruskal-Wallis test and post-hoc Dunn’s test with Holm’s multiple comparison adjustment were used). Only group 3 differed significantly from the other two groups, while no significant differences were observed between groups 1 and 2. Thus, based on the statistical differences between the groups, we pooled groups 1 and 2 together, which resulted in two age cohorts: young (18–59 years old; *M* = 39.3, *SD* = 13.1; *N* = 69) and elderly (60+ years old; *M* = 69.7, *SD* = 6.6; *N* = 37).

#### Main PWA group

All PWA except one were recruited at the Center for Speech Pathology and Neurorehabilitation, Moscow, Russia. None of them had history of neurodegenerative disorders or substance abuse. There were 85 participants in the PWA group (see Table 1 for demographic characteristics); 44 participants were in the young (age: *M* = 49.1, *SD* = 10.3) and 41 in the elderly age cohorts (age: *M* = 66.8, *SD* = 4.8). The level of formal education varied from incomplete secondary school (8 years) to a university degree (typically 15 years), with the majority of the participants (58.8%) having some form of higher education degree (12–15 years). Most participants were premorbidly right-handers (*N* = 80), one was left-handed, one–a retrained left-hander, and three were ambidextrous.

All the PWA had speech and language deficits due to focal brain damage of various etiology confirmed by a CT or an MRI scan. Most participants had damage to the left hemisphere (*N* = 82); two had bilateral damage; one had combined damage to the left hemisphere and the vertebrobasilar area. The majority of the PWA (*N* = 77) had a single or recurrent stroke (ischemic or hemorrhagic); six had traumatic brain injury (TBI); one had impairments due to tumor resection (meningioma); and one had a complex etiology (TBI + infection + toxic). All were diagnosed with aphasia after a standard clinical examination by a certified speech pathologist or neuropsychologist. According to medical histories, all participants had normal or corrected-to-normal vision; seven participants had decreased hearing acuity.

Participants with aphasia also had a range of concomitant impairments, including deficits in speed of processing, attention, working memory, and motor control as indicated in their medical histories. All of these noted cognitive deficits were secondary and minor relative to their primary aphasia diagnosis and these deficits did not compromise informed consent, the validity of the assessment or the interpretation of outcomes. No patients in the current sample had visual agnosia or neglect, one participant had hemianopsia, however, this was accommodated during testing by placing the stimuli in the preserved field of view. For generalizability purposes in this project, it was important to have a varied PWA sample, representative of the target population in a real-life clinical setting. Accordingly, we opted for maximally broad inclusion criteria, without compromising validity of findings.

Depending on the type of aphasia the patient was classified as either having a fluent or a non-fluent type of aphasia, or determined non-classifiable in the cases when the type of aphasia could not be identified unambiguously (see Table 2) according to the aphasia classification by Luria [3, 4, 30]. Specifically, individuals diagnosed with motor (roughly corresponding to Broca’s aphasia in the Boston classification) and/or dynamic (transcortical motor) aphasia based on the comprehensive neuropsychological evaluation were grouped into the “non-fluent” group, while individuals with sensory (Wernicke’s) and/or acoustic-mnestic (anomic) aphasia were included in the “fluent” group (see [3, 4, 30] for a more detailed discussion of the correspondence between various aphasia types in the different classification systems). Here we would like to emphasize that the assignment of the “non-fluent” and “fluent” labels was not grounded on the performance on a particular fluency task, but on the qualitative division between two major aphasia syndromes. This distinction has been acknowledged in many different aphasia classifications and remains widely accepted [31].

**Table 2:** Fluency groups determined according to Luria’s classification of aphasia in the main PWA group.

	Age group		
Fluency groups	*Young*	*Elderly*	*Total*
***Fluent***	13	23	36
***Non-fluent***	23	13	36
***Non-classifiable***	7	4	11
***NA***	1	1	2

*Note*. For NA cases, no full record of neuropsychological examination is available.

Additionally, for all the PWA except one individual a clinically established comprehensive language battery, the ASA [5] (described in detail in the Introduction) was also administered, which provided an overall score reflecting general severity of language impairment.

#### Inter-rater PWA group

For the analysis of inter-rater reliability, we randomly selected 20 individuals from the main aphasia group who completed all the subtests (see Table 1 for participants’ demographic characteristics). Because comprehension subtests were scored automatically (see description below), we evaluated inter-rater reliability only for the subtests targeting expressive language (repetition and production).

#### Test-retest PWA group

For evaluation of test-retest reliability it was essential to eliminate the potential effect of treatment on changes in RAT scores. Since the main PWA group was recruited from the in-patient department of the Center for Speech Pathology and Neurorehabilitation, where they were receiving intense speech-language therapy for several hours a day, an improvement in scores between two testing sessions would be expected for them. Accordingly, to evaluate test-retest reliability, the RAT was administered on two separate occasions to 20 different individuals with chronic aphasia (not included in the main aphasia group) prior to their admission to the Center for Speech Pathology and Neurorehabilitation. Here, specifically to rule out spontaneous recovery, only individuals at least 6-months post-onset were recruited. The two testing sessions were on average 25.3 days apart (*range* = 14–47; *SD* = 9) with no treatment provided between sessions. Due to a technical error, data for three subtests (discourse comprehension, sentence production, and discourse production) were not recorded. To evaluate test-retest reliability of the abovementioned subtests, we recruited additional 20 PWA with chronic aphasia (again, not included in the main aphasia group) and tested them on these three subtests on two separate occasions (two testing sessions were on average 9.3 days apart; *range* = 4–18; *SD* = 4.1). Demographic characteristics for these two PWA groups are presented in Table 1.

All participants (PWA and NHI) gave their informed consent to participate in the study. NHI participants signed a written consent form; PWA participants provided spoken consent and were informed by the researchers that they can freely decline or stop participating in the study at any time without compromising their eligibility to receive appropriate treatment from the Center. The study was approved by the Committee on Interuniversity Surveys and Ethical Assessment of Empirical Research of the HSE University.

### Rationale and development of the RAT

#### General structure of the test

The RAT is composed of three main parts: *auditory comprehension*, *repetition*, and *oral production*. Within each domain, individual subtests target processing at different language levels including phonological, lexical-semantic, syntactic, and discourse. *Auditory comprehension* is assessed at different levels of linguistic analysis with the following subtests: nonword discrimination, auditory lexical decision, single word comprehension of nouns and verbs, sentence comprehension, and discourse comprehension. This hierarchical set of subtests allows to determine the language level(s) at which comprehension breaks down. The *repetition* subtests include: nonword, word, and sentence repetition–and are designed to evaluate integrity of sublexical and lexical pathways for repetition, along with auditory short-term memory capacity. The *oral production* subtests encompass naming of objects and actions, sentence production with syntactic priming, and picture-elicited discourse production. This selection of subtests helps to distinguish between impairments in production at the lexical-semantic and syntactic levels, and to assess these abilities cumulatively under relatively natural conditions (in discourse production), enabling detection of mild residual deficits in expressive language abilities.

Within each subtest, (psycho)linguistic variables known to impact language processing in PWA are systematically manipulated to ensure inclusion of items of varying difficulty and to provide detailed information about intact and impaired components of the language system. Where applicable, item selection was based on relevant psycholinguistic parameters of the verbal and pictorial stimuli (imageability, age of acquisition, name agreement, image agreement, object / action familiarity, visual complexity: http://en.stim-database.ru, [32–34]; lemma frequency [35]). The manipulation of critical (psycho)linguistic variables helps to dissociate PWA’s difficulties at specific linguistic levels more accurately. Additionally, matched stimuli across some of the subtests allow for a direct comparison of PWA’s performance across tasks and domains. Specifically, the items in the single word comprehension subtests were matched with the items in the naming subtests on a number of psycholinguistic parameters. Additionally, the sentence comprehension and production subtests employed similar syntactic constructions. See Table 3 for a brief description of the subtests’ design and supporting information for a comprehensive account and a detailed rationale behind their construction (see S1 File).

**Table 3:** Description of the RAT subtests.

DOMAIN	SUBTEST (LEVELS EVALUATED)	TASK (# ITEMS)	FACTORS MANIPULATED	EXAMPLE IN RUSSIAN (ENGLISH TRANSLATION; TYPE OF STIMULI)
**AUDITORY COMPREHENSION**	Nonword discrimination (phonological)	Listen to pairs of nonwords and identify whether they are the same or different n = 22	• Consonant distinctive features (manner, place of articulation, voicing, palatalization) • Target position (initial, final) • Syllabic structure (VC, CV, CVC, CCVC, CVCC, CCVCC)	• “ро” /ro/—“ло” /lo/ (manner of articulation, initial, CV) • “друф” /druf/—“труф” /truf/ (voicing, initial, CCVC)
	Lexical decision (lexical)	Listen to sound strings and identify which of them are real words n = 24	• Lexical status: words (only low frequency, concrete words) vs. nonwords based on real words • Length (2 vs. 3 syllables long)	• “кенгуру” (kangaroo) (word) • “дловарь” /dlɐˈvar^j^/ (nonword based on the word “словарь” /slɐˈvar^j^/ (dictionary); analogous to dictionary → mictionary)
	Single word comprehension: nouns & verbs (lexical-semantic)	Listen to the word and match it with one out of four pictures Nouns: n = 24 Verbs: n = 24	• Part of speech (nouns vs. verbs) • Frequency • Relationship between the target and the distractors (phonological, semantic, unrelated) • Items in noun and verb comprehension matched on psycholinguistic parameters • Matched on the same psycholinguistic parameters with the items in the naming subtest	• Nouns: “ракета” (rocket—*target*)– “космонавт” (astronaut—*semantic distractor*)– “ракетка” (racket—*phonological distractor*)– “мяч” (ball—*unrelated*) • Verbs: “петь” (sing—*target*)– “танцевать” (dance—*semantic distractor*)– “пить” (drink—*phonological distractor*)– “есть” (eat—*unrelated*)
	Sentence comprehension (syntactic)	Listen to the sentence and match it with one out of two pictures n = 24	• Construction type (simple active constructions, subject and object relative clauses, prepositional constructions) • Word order (canonical vs. noncanonical) • Semantic reversibility (reversible vs. irreversible)	• “Где мальчик спасает девочку?” where boy.NOM rescue.PRS.3 girl.ACC (Where is the boy rescuing the girl?) (simple, canonical, reversible) • “Где мальчик кладет в сумку колбасу?“ where boy.NOM put.PRS.3 in bag.ACC sausage.ACC (Where is the boy putting the sausage in the bag?) (prepositional, noncanonical, irreversible)
	Discourse comprehension (discourse)	Listen to a story and verify a set of statements about events/details n_texts_ = 1 n_statements_ = 16 (8 pairs, statements within a pair refer to the same story element)	Type of statements: • relation to the story (plot-focused vs. detail-focused) • type of information (explicit vs. implicit) • veracity (true vs. false)	[Pair 1] • “Наташа приготовила еду, чтобы взять ее на озеро” (Natasha made food to take it to the lake) (plot-focused, explicit, true) • “Наташа приготовила еду, чтобы пригласить в гости соседей” (Natasha made food to invite the neighbors over) (plot-focused, explicit, false) [Pair 2] • “На берегу озера растет большое дерево” (There is a big tree on the shore of the lake) (detail-focused, implicit, true) • “На берегу озера совсем нет деревьев” (There are no trees on the shore of the lake) (detail-focused, implicit, false)
**REPETITION**	Nonword repetition (phonological)	Listen to nonwords and repeat them back n = 24	• Wordlikeness (high vs. low) • Length (1, 3 and 5 syllables long) • Number of articulatory switches, defined as the number of transitions between primary place of articulation of any two adjacent consonants (0 to 5)	• “мариация” /mər^j^ɪatsᵻjə / (based on the word “вариация” /vər^j^ɪatsᵻjə /(variation), analogous to variation → mariation) (high-wordlikeness, 5-syllable long, 2 articulatory switches) • “исхофа” /ɪshofə/ (low-wordlikeness, 3-syllable long, 3 articulatory switches)
	Word repetition (phonological, lexical)	Listen to words and repeat them back n = 24	• Frequency (high vs. low) • Length (1, 3 and 5 syllables long) • Number of articulatory switches (0 to 5)	• “территория” (territory) (high-frequency, 5-syllable long, 1 articulatory switch)
	Sentence repetition (lexical-semantic)	Listen to sentences and repeat them back n = 12	• Sentence length (3 vs. 6 content words) • Frequency of lexical items (high vs. low)	• “Машина опять не работает” (The car is not working again) (short, high-frequency condition) • “Капризная барышня критикует чудной цветочный орнамент” (The capricious baroness criticizes the intricate floral ornament) (long, low-frequency condition)
**ORAL PRODUCTION**	Naming: objects & actions (phonological, lexical-semantic)	Name objects or actions depicted in the picture Objects: n = 24 Action: n = 24	• Part of speech (nouns vs. verbs) • Frequency (high vs. low) • Items in object and action naming matched on psycholinguistic parameters • Matched on the same psycholinguistic parameters with the items in the single word comprehension subtest	• Objects: “кровать” (bed) • Actions: “вырезать” (cut out)
	Sentence production (lexical-semantic, syntactic)	Describe the picture according to the provided spoken model (syntactic priming paradigm) n = 24	• Construction type (simple active constructions, subject and object relative clauses, prepositional constructions) • Word order (canonical vs. non-canonical) • Semantic reversibility (reversible vs. irreversible)	• [Prime] “Невесту везет жених” bride.ACC give ride.PRS.3 groom.NOM (The bride is given a ride by the groom), [Target] “Дедушку кормит девочка” grandfather.ACC feed.PRS.3 girl.NOM (The grandfather is fed by the girl) (simple, non-canonical, reversible) • [Prime] “Девушка кладет авоську в сумку” gorl.NOM put.PRS.3 string bag.ACC in bag.ACC (The girl is putting the string bag in the bag) [Target] “Девочка кладет бочку в коробку” girl.NOM put.PRS.3 barrel.ACC in box.ACC (The girl is putting the barrel in the box) (prepositional, canonical, reversible)
	Discourse production (lexical-semantic, syntactic, discourse)	Produce a story based on the presented picture with exposition, climax and resolution. n = 1		

All visual stimuli accompanying different RAT subtests consisted of black-and-white line drawings. For single-word comprehension and naming subtests, pictures were taken from the Database of Russian Verbs and Nouns [32–34]. The visual stimuli for the sentence comprehension and production subtests, and the picture for the discourse production subtest were specially created for the RAT by the same artist. Auditory stimuli for all the subtests were recorded in a studio by a professional male speaker.

#### Implementation of the RAT on a tablet

We created a custom Android-based tablet application for automatic stimuli presentation and response registration for all RAT subtests. The application was developed using Java SE 8 programming language and can be installed on touch-screen tablets running an Android OS (4.2 and higher). Performance on the comprehension subtests (selection of a visual stimulus matching the auditory stimulus or selection of a yes/no response) is registered and scored automatically by the application. Reaction times are also registered for comprehension subtests; however, they were not analyzed within the present study and are not currently used for diagnostic purposes. Response accuracy and reaction time for each trial for comprehension subtests can be downloaded as.csv files for subsequent analysis. Oral responses to repetition and oral production subtests are recorded automatically for each trial separately and can be downloaded as .3gp files and analyzed manually.

### Administration of the RAT

The RAT was presented on the Samsung Galaxy Tab A (model SM-T585) or Tab 4 (model SM-T531) tablets. Both tablet models had a screen size of 10.1 inches and the following screen resolution: 1920 x 1200 and 1280 x 800 pixels, respectively. While the test can be administered and scored in a traditional paper-and-pencil format (a paper-copy version of the stimulus cards and the scoresheets in Russian are available at– https://www.hse.ru/en/neuroling/research/rat/), the reported data were collected using the electronic version of the test. No special tablet use skills, such as dragging or swiping, are required on the participant’s part to complete the tablet-based version of the RAT, and all navigation between subtests and items (except in the comprehension subtests, see below) was done by the examiner. The whole test took 1–2 hours to administer (depending on the patient’s aphasia severity). The NHI group completed the test in one session; the PWA required 1–3 testing sessions (depending on the fatigue levels) within the same week to complete the test.

All the subtests started with presentation of written instructions on the tablet screen (see S1 File for individual subtest instructions). The examiner read them aloud and provided additional explanations if necessary. The instructions were followed by several practice items (typically three). Here, the examiner ensured that the participant understood all the instructions, repeating the practice items multiple times and providing feedback and clarifications, if needed. After clarifying all the questions, the actual test items were presented. For all the subtests, the visual stimulus appeared first and was followed (where applicable) by the auditory stimulus with a 2-second delay. For the comprehension subtests, the next trial was automatically triggered upon response selection. For the repetition and production subtests, the examiner manually advanced to the next trial once the participant provided a verbal response. Participants’ responses on the repetition and production subtests were not time-limited, although the examiner urged the participants to provide a response in case of an abnormally prolonged hesitation, i.e., when there was a pronounced delay in responding to a test item relative to responses provided previously by the same patient. If they failed to do so, the examiner proceeded to the next item and the item was marked as no response (incorrect). A single repetition of the test item upon request was allowed and incurred no penalty. No meaningful cues from the examiner were permitted. Sometimes for psychological reasons (e.g., to minimize a participant’s frustration), it was necessary to repeat the stimulus item multiple times or provide a cue; however, subsequent responses were not scored, and the item was marked as incorrect. In cases when the patient gave continually erroneous responses, the examiner was instructed to still complete administration of the subtest. Only if the patient persistently, for several items in a row provided no responses, the remaining items were marked as incorrect.

### Scoring guidelines

For the comprehension subtests, each response was automatically registered as correct or incorrect. As stated above, a single repetition of the item was allowed. The correct items gained a score of 1, incorrect–a score of 0; the total raw score for each subtest was the sum of the item scores.

In the current study, the recorded verbal responses in the production and repetition subtests were downloaded from the tablet to a computer, transcribed and scored manually according to subtest specific criteria (see below). In terms of real-life procedures, clinicians can fully rely on the application for scoring by replaying the individual responses on a trial-by-trial basis on the tablet after the test is administered and scoring them there. Alternatively, they can perform the scoring in real time and score responses while the test is being administered in the paper protocol. In either case, a full transcription is unnecessary for clinical administration of the test. Self-corrections in the repetition and production subtests entailed no penalty, but only the last verbal response was scored (even if the original response was correct). Additionally, verbal responses were not marked down for typical dysarthric distortions.

In the nonword and the single word repetition subtests, a score of 1 was given for a correctly repeated item (nonword/word), 0.5 for a phonological paraphasia (when more than 50% of the target word is spared, and the target is still recognizable), and 0 for all other types of errors. In the sentence repetition subtest, each word in the sentence was scored separately in a similar fashion. For each correctly repeated content and functional word in the sentence, 1 point is given. Phonological paraphasias and word form errors are scored as 0.5, while omissions and other errors are given a score of 0. Word order changes, repetitions, omissions, and insertions that altered the word order in the sentence incur an order penalty of 1 (irrespective of the number of such errors). Then the score for each sentence is calculated as the sum of the word points minus the order penalty. The total raw score for each subtest was the sum of the item scores.

In the naming subtest, a correct response was scored as 1, and all error types as 0. In the sentence production subtest, each sentence was evaluated according to four criteria: consistency with the prime, grammaticality, lexical-semantic adequacy, and other aspects of phrase appropriateness. A score of 1 was given if a criterion was met, and 0 if it was not. The sum of the scores on the four criteria constituted the score for a particular item; the total raw score was the sum of the item scores. Performance on the discourse production subtest was rated on a 5-point scale (with higher score corresponding to better performance) on each of the four criteria: fluency, grammatical complexity, paraphasias, and informational content. The total raw score for this subtest was the sum of the scores on the four scales.

Finally, subtest accuracy was defined as the percentage of correct responses out of the maximum possible score for all the scored items. As a measure of overall language impairment, we computed the General Aphasia Quotient (GAQ). It is used to determine presence and severity of aphasia and is calculated as an average of total percentage scores for all the subtests. Thus, it ranges from 0 to 100%. GAQ can be calculated only if total scores of all the subtests are available (i.e., all the subtests were administered to the participant, fully or partially).

More comprehensive explanation of the scoring procedures for each subtest are provided as supporting information in the detailed description of the RAT subtests (see S1 File). Prospective users of the RAT are encouraged to follow the same administration and scoring guidelines to ensure the validity of results.

### Data analysis

We established the following psychometric properties of the test based on the NHI and PWA data (all statistical analyses were done in R [36] and figures were drawn in ggplot2, ver. 3.3.2 [37]):

1. *Cut-off values* indicating impairment for both individual subtests and the overall score (GAQ) were determined. For individual subtests, the 5^th^ percentile of the respective control group was used as the cutoff. To determine a cut-off value for the presence of aphasia, the Receiver Operating Characteristic (ROC) curve analysis was performed using the GAQ of the NHI and the PWA groups. These analyses support the use of the test to identify a language impairment in individuals with brain damage relative to healthy controls.
2. *Severity ranks* (mild, moderate, severe) for each subtest and GAQ were established based on percentile ranges of the PWA group. This analysis offers evidence that the RAT can measure impairments of varying severity in different domains and the test can be used to quantify overall aphasia severity.
3. *Concurrent validity* of the test was ascertained by correlating the GAQ based on the RAT with the overall scores on the ASA [5]. This further supports the claim that the RAT can capture the full spectrum of aphasic language disorders.
4. *Construct validity* was evaluated based on correlation patterns between the RAT subtests. These findings provide further support that the test offers a multidimensional evaluation of linguistic strengths and weaknesses across multiple language domains.
5. *Inter-rater and test-retest reliability* of the test was determined using inter-class correlations. These estimates offer evidence that the RAT measures language deficits reliably over time and irrespective of the rater, which is important for all proposed applications of the test.

## Results & discussion

### Overall performance of the NHI and PWA groups

**Fig 1:**
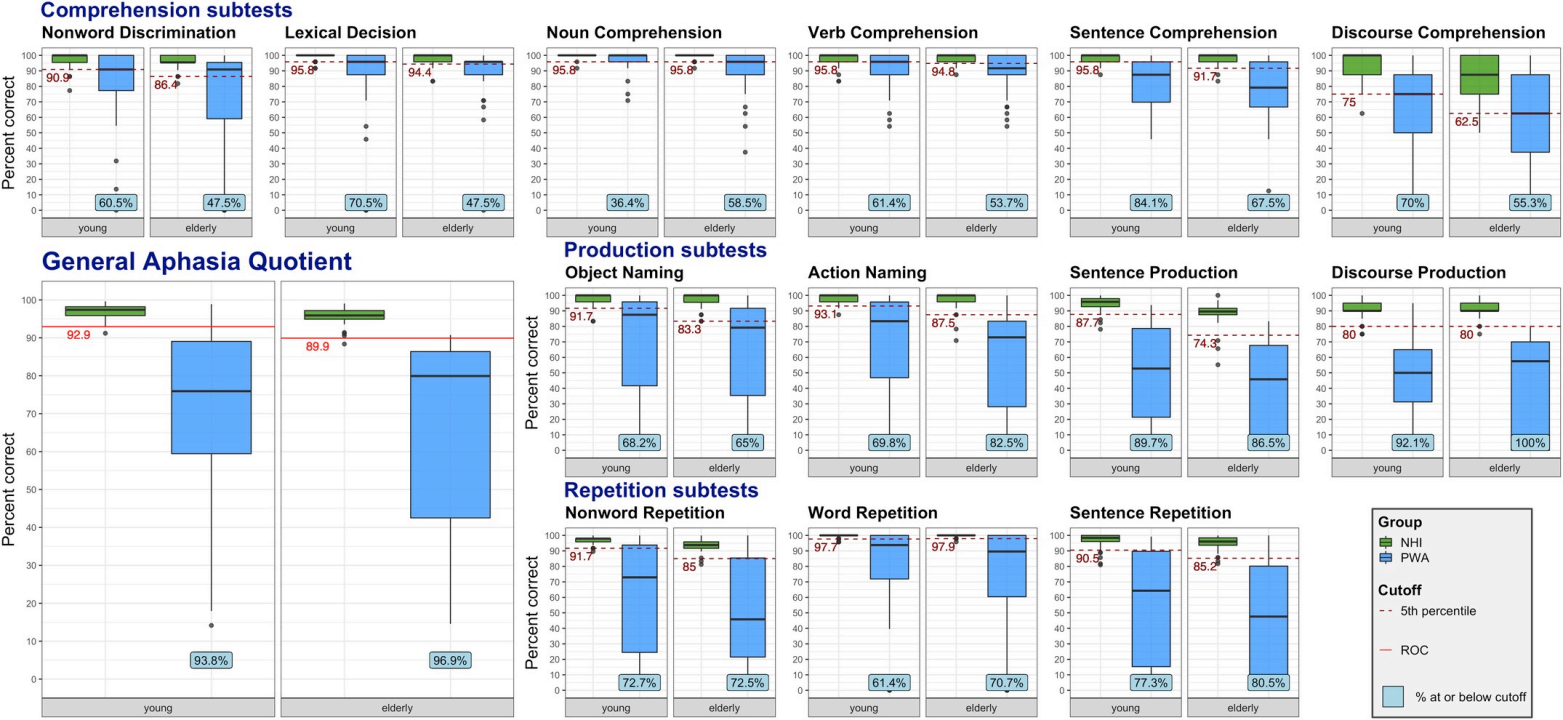
Accuracy subtest scores and the General Aphasia Quotient (GAQ) for the control group of neurologically healthy individuals (NHI) and the main group of people with aphasia (PWA) for each age cohort. The box represents the interquartile range, with the central line marking the median. The whiskers denote the largest/smallest values within 1.5 times the interquartile range above/below the 75^th^/25^th^ percentile. Values falling outside of that range are shown as black points. Red lines indicate cutoff thresholds: dashed for each subtest, representing the 5^th^ percentile of the control group, and solid for the GAQ cutoff, determined according to Receiver Operating Characteristic (ROC) curve analysis. The cyan box with percentages represents percent of PWA performing in the impaired range (at or below cutoff for normal performance for a given subtest).

Descriptive statistics for each subtest scores and GAQ for the NHI and PWA for both age cohorts are provided as supporting information (see S1 Table). Additionally, accuracy scores for each subtest for the two participant groups across the two age cohorts are presented as boxplots in Fig 1.

The distribution of scores clearly demonstrates that the NHI group performed near ceiling in most subtests, apart from Discourse Comprehension, Discourse Production, and Sentence Production. This lack of a ceiling effect in these specific subtests could be due to the complexity of these tasks and participants’ variability in non-linguistic pragmatic skills (Discourse Comprehension, Discourse Production) and memory (Discourse Comprehension, the priming component of Sentence Production). Additionally, the two age cohorts in the NHI group performed comparably, except for subtests targeting phonological processing and/or memory (independent-samples two-tailed Welch t-tests: Discourse Comprehension, *t* = -3.47, *p* = .001; Nonword Repetition, *t* = -4.17, *p* < .001; Sentence Production, *t* = -4.01, *p* < .001; and borderline significant for Sentence Repetition, *t* = -3.01, *p* = .0039), with significance value Bonferroni-corrected to *p =* .05/13 = .0038. This reflects typical patterns observed in the aging population due to increasing sensory deficits, declining working memory and general slowing [38, 39].

Performance of individuals with aphasia was more variable, especially for repetition and production subtests, reflecting different language profiles and deficit severity in the main PWA group (Fig 1). Out of the whole aphasia group (*N* = 85), 68% (*N* = 64) completed all the subtests of the RAT.

As expected, PWA obtained significantly lower scores across all subtests compared to the NHI group in both age cohorts (based on independent-samples two-tailed Welch t-tests with significance value Bonferroni-corrected to *p* = .05/26 = .0019*)*. Two exceptions were Noun Comprehension in the young group and Lexical Decision in the elderly group, where the difference trended towards significance (*p* = .003 in both cases). To determine the effect of fluency, an independent-samples two-tailed Welch t-test was performed between fluent (*N* = 36) and non-fluent groups (*N* = 36). No significant differences (*p* > .05) in any subtest were revealed, underscoring the prevalence of various linguistic deficits irrespective of aphasia group and the importance of detailed linguistic diagnostics.

Additionally, we wanted to verify that documented decreased hearing acuity in PWA was adequately corrected and that it was not influencing performance on subtests where peripheral auditory function is particularly critical, such as the Nonword Discrimination, Lexical Decision, and Single word Comprehension. We compared performance on those subtests between PWA with reported hearing acuity deficits (*N* = 7) and without (*N* = 78) using the Fischer’s exact test that allowed to establish whether the distribution above and below cutoff was similar in the two groups. Across all the four subtests of interest no significant differences between the two groups in the proportion of patients performing below the cutoff were observed verifying that hearing deficits did not influence performance on the subtests.

### Item analysis

We calculated item passing rates (average score for each item) based on the PWA data (see S3 Table for individual item passing rates for all subtest). Comprehension subtests had overall higher item passing rates (mean of item passing rates across comprehension subtests ranged between 0.77 and 0.94) compared to both repetition (0.51–0.75) and production (0.44–0.64) subtests, again reflecting the fact that subtests targeting expressive language abilities are on average more difficult for PWA. However, all subtests, except for Noun and Verb Comprehension subtests, comprised items sufficiently ranging in difficulty. Relative to other subtests, the Noun and Verb Comprehension subtests did show a narrower spread in item passing rates and generally higher values, signifying that single word comprehension abilities are the least impaired in aphasia.

Next, we calculated corrected item-total correlations (correlation between the item score and the total subtest score minus that item, computed using the psych package for R [40]) based on the PWA data (see S4 Table for individual item values). The high values (mean of corrected item-total correlations across subtests ranged between 0.36 and 0.86) and the observed spread reflected good overall coherence and at the same time sufficient discriminability of subtest items. Only a few items in the Noun and Verb Comprehension subtests, and one item in both Nonword Discrimination and Sentence Comprehension showed poor item discriminability (corrected item-total correlation below 0.2). The relatively lower values observed again for the Noun and Verb Comprehension subtests reflect the ceiling effects and limited spread in scores on the subtest items. Overall, the item analysis indicated a sufficient range of item difficulty across the RAT subtests and good consistency in test items. This suggests that the RAT is robustly sensitive to the full spectrum of aphasic language disorders.

### Establishment of cutoff values and the RAT’s sensitivity and specificity

Cutoff values were determined differently for the subtests and for the GAQ. To calculate subtest cutoffs, we first removed outliers in the NHI group, defined as subtest scores lower than 90 percent and more than three standard deviations away from the mean value of the respective age cohort. This minimized the influence of single aberrant values on the cutoff criteria. Overall, one to two outliers were removed in several subtests in both the young and the elderly NHI cohorts. Next, for each individual subtest, the 5^th^ percentile of the NHI group’s score was calculated separately for the two age cohorts, which was considered the cutoff for impaired performance (see Fig 1). Performance at or below cutoff was considered abnormal, since 95% of healthy controls without a language impairment scored higher (cf., CAT [24]). The only exception to this rule was the Noun Comprehension subtest for the young cohort. In this one subtest, the 5^th^ percentile equaled 100%, hence the cutoff for impaired performance was adjusted to the next score possible– 95.83% (23 out of 24 trials). Otherwise, even a score of 100% would be labeled as impaired. For percentages of PWA with abnormal performance on each subtest in two age cohorts see Fig 1. It should be emphasized that while a score at or below the cutoff indicates a deficit in a given task, it does not by itself imply presence of aphasia. The latter is ascertained based on the GAQ.

To determine the diagnostic cutoff for the GAQ, a ROC-curve analysis was performed separately for each age cohort using the pROC package [41]. A ROC graph is a visual representation of a classifier where true positive rate (sensitivity, i.e. classifying a PWA as a PWA) is plotted against false positive rate (1 –specificity, i.e., classifying an NHI as a PWA) and thus demonstrates the trade-off between the two. A ROC-curve is a step function where performances of the classifier for the same dataset but at different threshold values are plotted [42]. Based on the ROC-curve, a threshold (cutoff) value can be selected that optimizes both sensitivity (positive identification of those with aphasia) and specificity (correct negative identification of those without the disorder).

**Fig 2:**
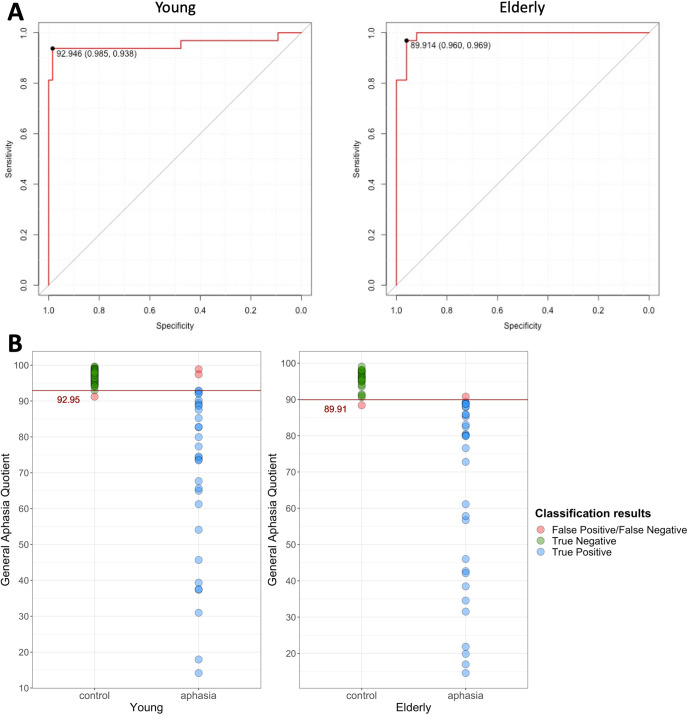
Determining the cutoff score for identification of aphasia. Panel A–Receiver Operating Characteristic (ROC) curves used to determine the General Aphasia Quotient (GAQ) cutoff score for the two age cohorts, with the x-axes flipped to plot sensitivity against specificity. The optimal threshold was selected to maximize the sum of sensitivity and specificity, as indicated by the black point on the graph. Panel B–classification accuracy for the two age cohorts according to the GAQ cutoffs.

In our case, two ROC-curves for two age cohorts were generated (see panel A in Fig 2). For convenience, the x-axes of the graphs were flipped to plot sensitivity against specificity. For this analysis, outliers were not removed, because the GAQ represents an average score across all subtests and is, therefore, not detrimentally impacted by aberrant values in individual subtests scores. Also, we wanted to account for a full range of possible performance in calculation of the aphasia cutoff. The optimal threshold was selected to maximize the sum of sensitivity and specificity [43]. A GAQ score at or below the cutoff indicates presence of aphasia.

Two separate cutoff values for the GAQ indicating presence of aphasia were determined: 92.95% for the young cohort and 89.91% for the elderly cohort (see Fig 1), reflecting minor challenges the elderly control group experienced with some of the subtests. The RAT demonstrated excellent diagnostic accuracy: sensitivity was .938 for the young and .969 for the elderly cohort, while specificity was .985 and .96, respectively. Three and two individuals were incorrectly classified as control instead of aphasia or aphasia instead of control group in the young and elderly cohorts, respectively (see panel B in Fig 2). The missed aphasic diagnoses included two patients with very mild residual aphasia and one patient with anomic aphasia of traumatic etiology. Overall, this substantiates high sensitivity and specificity of the RAT, with its diagnostic accuracy comparable to other tests that used ROC curve analysis to determine aphasia cutoff (cf., QAB [22]).

### Determination of impairment severity

We empirically defined three impairment severity ranks (mild, moderate, and severe) for each subtest and for the GAQ based on the PWA data. To find the relevant ranges, we first selected, for each subtest and for the GAQ, the PWA with abnormal performance at or below the cutoff. Then the two age cohorts were combined to increase sample size and to ensure that the calculated ranges were not influenced by our two age cohorts being unbalanced in terms of aphasia types (see Table 2 in the Methods section). Next, we divided the accuracy scores into three approximately equal ranges by calculating the 34^th^ and the 67^th^ percentiles of the subtest scores / GAQ values for these combined groups. Specifically, for both the RAT subtest scores and GAQ values the three severity ranks were defined as the following, where *X*_*i*_ is the individual participant’s accuracy score on a given subtest/GAQ:

- Mild: 67^th^ percentile ≤ *X*_*i*_ ≤ cutoff;
- Moderate: 34^th^ percentile < *X*_*i*_ < 67^th^ percentile;
- Severe: 0 ≤ *X*_*i*_ ≤ 34^th^ percentile.

**Fig 3:**
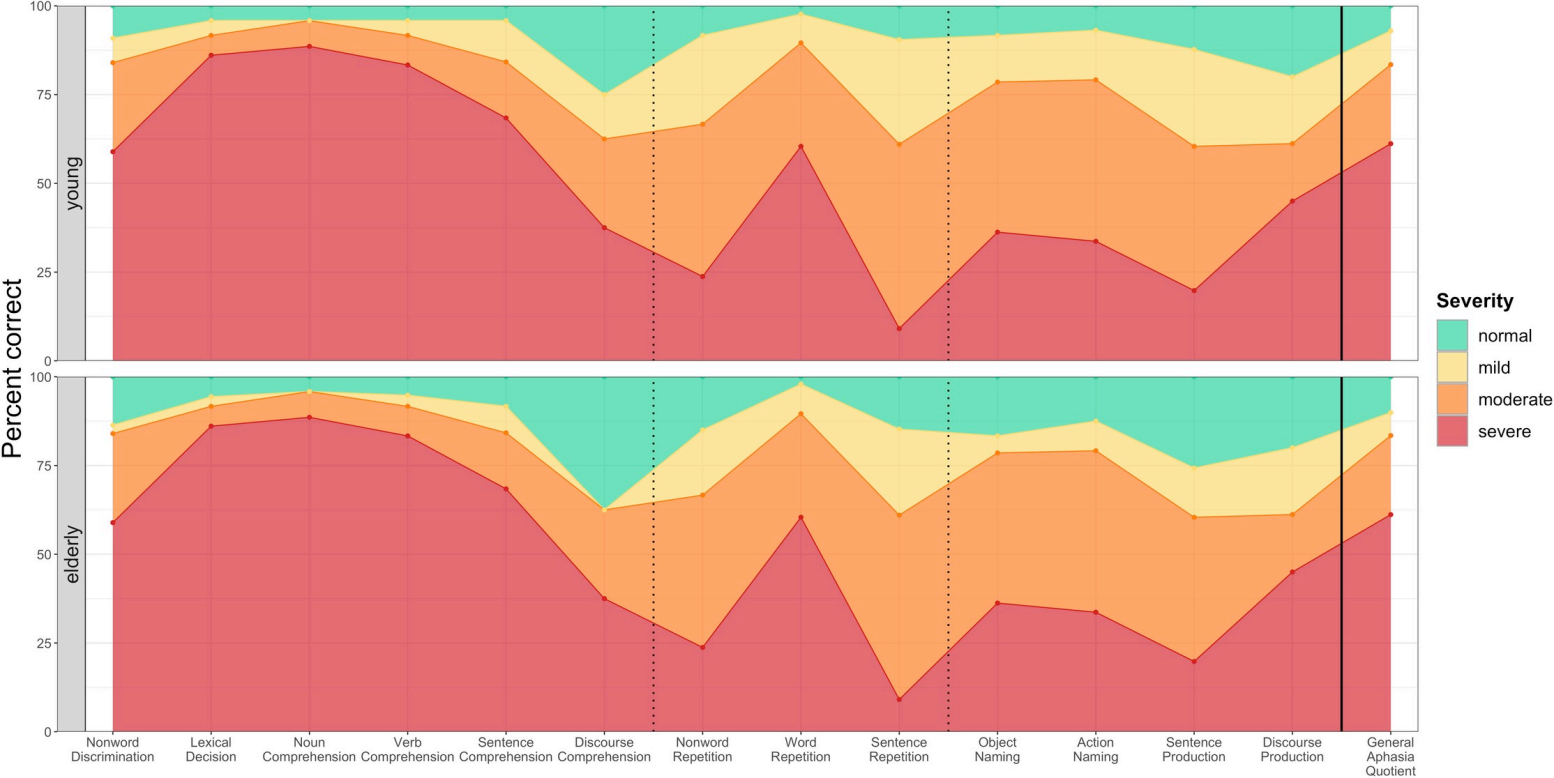
Severity ranks for subtest accuracy scores and the General Aphasia Quotient (GAQ) for the two PWA age cohorts.

Performance above cutoff was considered normal. See Fig 3 for severity ranges for subtest accuracies and the GAQ and supporting information for respective values (see S2 Table). Note that the cut-off for normal performance varies between the two groups (it is based on the 5^th^ percentile of the respective NHI cohort, except for Noun Comprehension–see previous section for more details), but the cut-offs for different severity ranks are identical for the two groups since we combined the two PWA age cohorts for this analysis.

As can be clearly seen from Fig 3, comprehension subtests, where PWA typically score higher (except for Discourse Comprehension), had narrower moderate and mild ranges, while for repetition and production tasks these ranges were more evenly distributed. This most likely reflects several factors: Comprehension subtests being easier due to their design (multiple-choice vs. open-ended questions for repetition/production), and comprehension abilities being more spared in aphasia in general. Accordingly, as the Discourse Comprehension subtest had a safeguard built in against guessing (i.e., to receive credit, participants had to respond correctly to two questions pertaining to the same part of the story), the severity ranges were less skewed towards the higher accuracy values. In other words, individual subtests were not (and probably could not) be equated for difficulty. This makes direct comparison of raw accuracy scores between subtests uninformative, making the appraisal of severity ranks across subtests more relevant. Also, from Fig 3 it can be inferred which subtests posed the most difficulty for our aphasia sample (where lowest accuracy scores corresponded to the threshold for severe level of impairment): Discourse Comprehension, Nonword Repetition, Sentence Repetition, Object and Action Naming, and Sentence Production.

Overall, we determined these severity ranks primarily for clinical purposes. Attributing a corresponding severity level of impairment to participant’s performance on each subtest allows clinicians to qualitatively compare different subtest scores and more clearly identify spared and impaired language domains. Additionally, it allows to track the patient’s progress between different time points. However, given the intercorrelations observed between different subtest scores (see section on Construct validity below) and typical low reliability of contrast scores (e.g., [44]), with the currently available data it is not possible to reliably differentiate genuine dissociations across linguistic levels and domains from measurement errors. Therefore, between-subtest differences cannot be appraised quantitatively and should generally be interpreted with caution, particularly in individual cases.

### Concurrent validity

**Fig 4:**
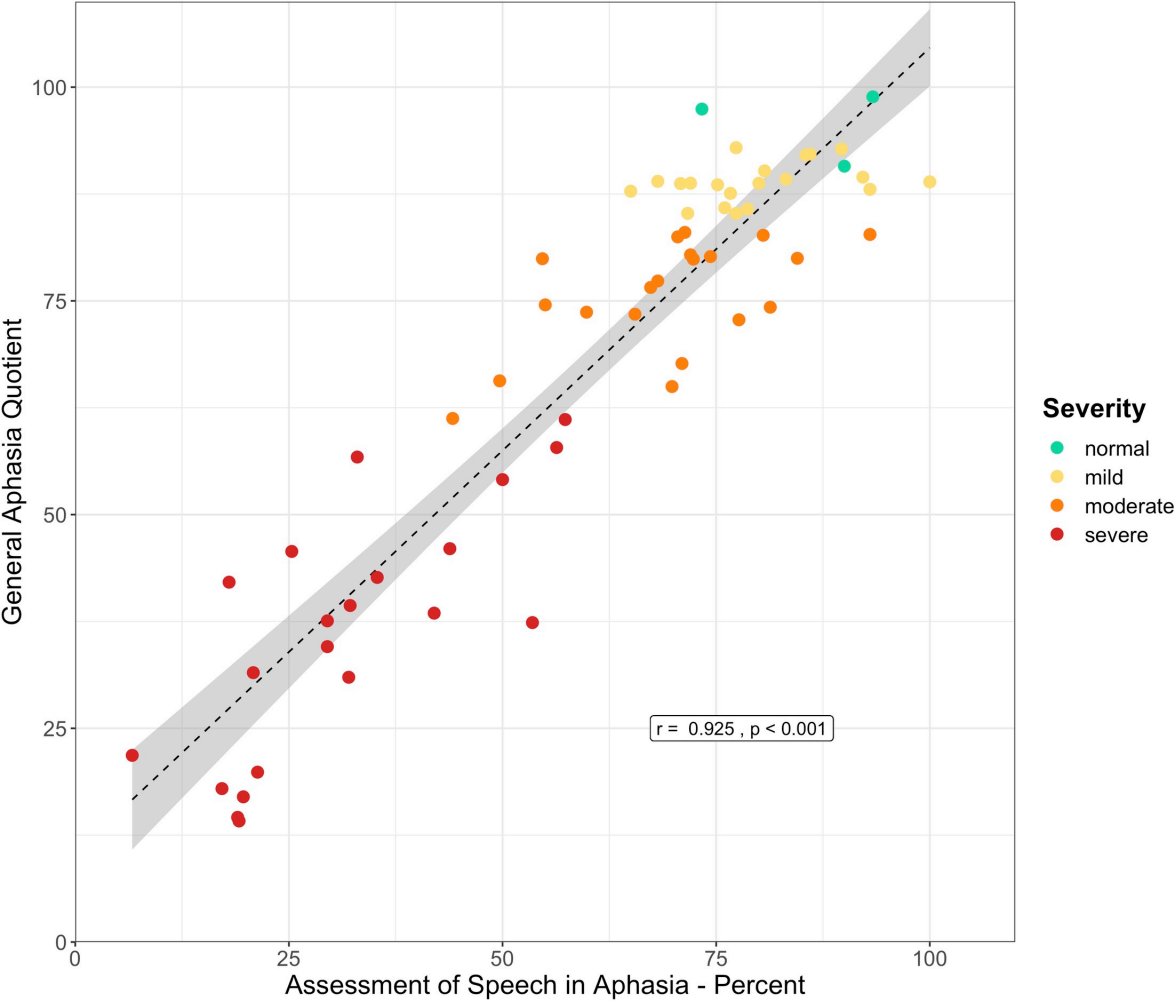
Correlation between the General Aphasia Quotient (GAQ) of the RAT and the total score on the Assessment of Speech in Aphasia (ASA), a widely used aphasia battery in Russian. Different colors indicate different severity ranks based on the GAQ.

Concurrent validity of the RAT was established by correlating the GAQ with the overall scores on the ASA [5]. A strong Pearson correlation was observed (*N* = 64, *r* = .925, *p* < .001, 95% CI [.879, .954]), substantiating the validity of the test (see Fig 4). However, the data also show that individuals with similar ASA total scores could obtain substantially variable GAQ scores, e.g., the same ASA score may correspond to normal, mild, or moderate impairment based on the GAQ score. On the other hand, it should also be noted that those participants identified as having mild aphasia according to the RAT demonstrated a range of scores on the ASA, but that range also fell into the interval for moderate-to-mild level of impairment according to the ASA classification (73% - 87%). While it is beyond the scope of the present paper to contrast the two tests in detail, these observations likely reflect different structures and scoring systems of the two tests. In the ASA there is greater impact of memory abilities on performance in the comprehension subtests (as participants have to comprehend strings of words of increasing length), and possibly inter-rater reliability issues with scoring of sentence and discourse production tasks (reliability of the ASA is not established). At the same time the RAT covers additional language domains (repetition), includes further language comprehension tasks (Nonword Discrimination, Lexical Decision), and affords more fine-grained assessment of language production at the sentence and discourse levels, potentially providing a more detailed evaluation of language deficits in aphasia. Accordingly, while a large correlation between the two tests supports concurrent validity of the RAT, the observed spread in scores reinforces the claim that the RAT offers additional insights and provides a more differentiative evaluation of language deficits in patients with brain damage.

### Construct validity

**Fig 5:**
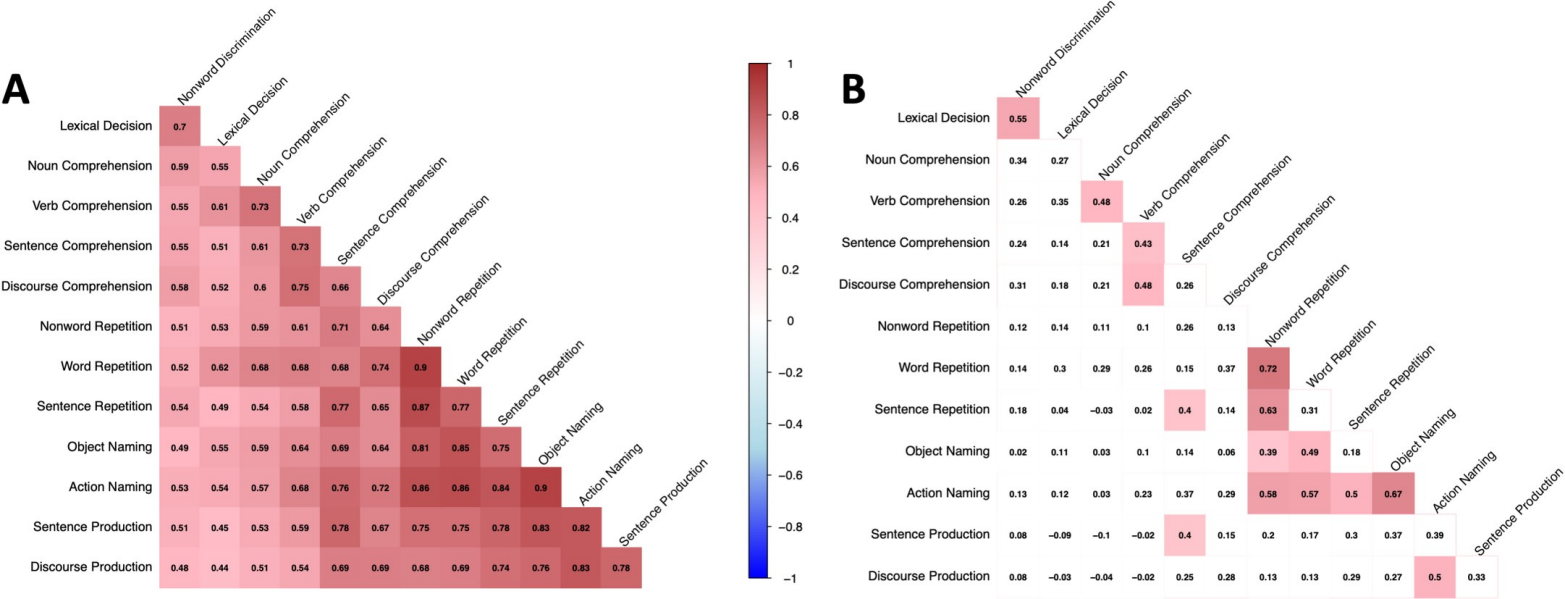
Correlation between the subtests’ scores. Panel A–simple Pearson correlations between the RAT subtests without accounting for aphasia severity. Panel B–partial Pearson correlations between the RAT subtests accounting for overall aphasia severity as measured independently by the total score on the Assessment of Speech in Aphasia (ASA). The plotted correlations are based on complete pairwise observations. The Bonferroni-corrected *p*-value equals .05 / 78 = 0.0006. Tiles with significant correlations are highlighted.

Next, we explored the inter-relationships between the RAT subtests. Notably, significant and strong Pearson correlations (Bonferroni-corrected for all pairwise comparisons) between all subtest scores were observed (see Fig 5A), as has been demonstrated previously for other language tests (QAB [22]; CAT [24]). We also performed a novel analysis that is not typically employed in aphasia batteries. We ran partial correlations (psych package for R [40]) between subtest scores accounting for aphasia severity as measured independently by the total score on the ASA [5]. Here, a more nuanced and domain specific pattern of correlations between subtests emerged after correcting for multiple comparisons (see Fig 5B). Only associations between subtests that measured similar underlying language abilities (within and across linguistic levels) continued to show a significant association, underscoring that the subtests are measuring what they intend to measure and are differentially sensitive to different underlying linguistic impairments.

Auditory comprehension subtests specifically targeting phonological and lexical levels (Nonword Discrimination and Lexical Decision) correlated only with each other and with no additional subtests. Single word comprehension subtests were also correlated. Interestingly, it was specifically verb comprehension that was found to be related to sentence and discourse level comprehension, supporting the idea of the role of the verb as a central sentence element. This correlation emerged even though most of the trials in the Sentence Comprehension subtest had syntactic but not semantic distractors. Accordingly, it is in line with the hypothesis that the verb’s grammatical properties (e.g., argument structure) are processed during comprehension and production of isolated verbs [45], linking the impairment at the single-verb and sentence levels. A similar relationship was observed between Action Naming and Discourse Production, with correlation between Action Naming and Sentence Production trending towards significance. Generally, comprehension subtests were not related to repetition and production subtests, except for the sentence-level tasks. Performance on the Sentence Comprehension subtest was related to both Sentence Repetition and Sentence Production subtests, suggesting that their performance may be relying on the same underlying processes, e.g., short-term/working memory and syntactic processing.

Repetition subtests remained highly correlated; it should be noted that specifically Nonword Repetition correlated strongly with Sentence Repetition likely indicating critical involvement of the phonological buffer in both tasks. As expected, Object and Action Naming subtests were related. Both naming subtests were also related to Nonword and Word Repetition subtests, but again it was specifically Action Naming that was related to the Sentence Repetition subtest and the Discourse Production subtest. Overall, the prominent relationships observed between performance on subtests targeting verb processing and sentence/discourse level tasks highlights the importance of assessing verb comprehension and action naming in aphasia. Future studies are needed to explore these differential association patterns in greater detail.

### Internal reliability

We estimated internal reliability of subtest scores using Cronbach’s alpha [46] calculated with the psych package for R [40]. While the Cronbach’s alpha has limited application for cases when the underlying construct is multi-dimensional [47], as is the case with most language subtests, it can provide at least an approximate lower-bound estimate of reliability even when tau-equivalence (i.e., the same true score for all test items, or equal factor loadings of all items in a factorial model) is violated. Internal reliability of RAT subtest scores was good (Noun and Verb Comprehension, Sentence Comprehension) to excellent (Nonword Discrimination, Lexical Decision, all repetition and production subtests), based on the criteria outlined by Koo and Li [48]. Thus, this provides preliminary evidence on the consistency of the items comprising the different subtests.

### Inter-rater reliability

Inter-rater reliability for repetition and production subtests was calculated based on 20 evaluations of PWA responses (the inter-rater PWA group, see Table 1 in the Methods section). Each subject’s response was evaluated independently by two trained raters according to the detailed scoring instructions. Inter-rater reliability was not computed for the comprehension subtests since those subtests are scored automatically by the tablet-based application. The intraclass correlation coefficients, absolute agreement (ICC, type A-1) for each subtest were calculated using package irr [49] and are shown in Table 4. The ICCs ranged from .833 (Discourse Production) to .994 (Object Naming). These ICC values indicate excellent inter-rater reliability for all subtests except Discourse Production, according to the criteria defined by Koo and Li [48]. The relatively lower (although still good) reliability of the Discourse Production subtest is a consequence of the more subjective nature of the scoring system for this task and is comparable to (or even exceeds) the inter-rater reliability values reported for other discourse rating scales [50]. No significant differences were observed between ratings provided by the two raters (paired-sample t-tests, *p* > .05). Overall, these results demonstrate that with the provided scoring instructions, the RAT can be reliably scored by trained clinicians.

**Table 4:** Reliability estimates of the RAT subtests: internal reliability estimated with Cronbach’s alpha, inter-rater and test-retest reliability based on the intraclass correlation coefficients, absolute agreement (ICC, type A-1).

	*Internal reliability*		*Inter-rater reliability*				*Test-retest reliability*			
*RAT subtest*	*Cronbach’s alpha*	*95% CI*	*ICC*	*95% CI*	*Rater 1*, *mean score*	*Rater 2*, *mean score*	*ICC*	*95% CI*	*Time 1*, *mean score*	*Time 2*, *mean score*
***Nonword Discrimination***	0.951	[0.936, 0.966]	-	-	-	-	0.675	[0.242, 0.868]	79.8%	88.0%
***Lexical Decision***	0.946	[0.929, 0.963]	-	-	-	-	0.777	[0.526, 0.905]	94.4%	95.6%
***Noun Comprehension***	0.841	[0.794, 0.888]	-	-	-	-	0.205	[-0.249, 0.585]	97.5%	98.3%
***Verb Comprehension***	0.797	[0.736, 0.858]	-	-	-	-	0.472	[0.043, 0.753]	95.0%	94.2%
***Sentence Comprehension***	0.838	[0.79, 0.887]	-	-	-	-	0.807	[0.581, 0.919]	85.2%	82.7%
***Discourse Comprehension***	0.83	[0.776, 0.885]	-	-	-	-	0.784	[0.53, 0.909]	70.0%	76.9%
***Nonword Repetition***	0.979	[0.973, 0.985]	0.987	[0.967, 0.995]	64.9%	66.4%	0.938	[0.849, 0.975]	71.5%	70.9%
***Word Repetition***	0.983	[0.978, 0.988]	0.968	[0.923, 0.987]	87.0%	86.0%	0.944	[0.865, 0.977]	86.0%	87.1%
***Sentence Repetition***	0.974	[0.966, 0.982]	0.997	[0.992, 0.999]	53.8%	53.5%	0.955	[0.893, 0.982]	60.4%	58.3%
***Object Naming***	0.964	[0.953, 0.975]	0.994	[0.985, 0.998]	77.6%	78.3%	0.937	[0.832, 0.976]	65.8%	70.0%
***Action Naming***	0.964	[0.953, 0.975]	0.975	[0.928, 0.991]	73.4%	75.8%	0.878	[0.716, 0.95]	62.6%	63.3%
***Sentence Production***	0.981	[0.976, 0.987]	0.965	[0.915, 0.986]	48.1%	49.7%	0.971	[0.925, 0.988]	57.3%	59.8%
***Discourse Production ^a^***	-	-	0.833	[0.609, 0.932]	57.0%	62.5%	0.71	[0.394, 0.875]	75.5%	75.2%
***GAQ-proxy-1 ^b^***	-	-	-	-	-	-	0.968	[0.923, 0.987]	79.8%	80.8%
***GAQ-proxy-2 ^c^***	-	-	-	-	-	-	0.939	[0.846, 0.976]	67.6%	70.6%

Note.

**a–**Cronbach’s alpha was not computed for Discourse Production as it only has one item.

**b–**GAQ-proxy-1 is calculated by averaging the subtests completed by the first Test-retest PWA group and comprises all the RAT subtests, except Sentence Production, Discourse comprehension, Discourse Production.

**c**–GAQ-proxy-2 is calculated by averaging the subtests completed by the second Test-retest PWA group and includes Sentence Production, Discourse comprehension, and Discourse Production subtests.

### Test-retest reliability

Test-retest reliability was calculated based on 20 additional individuals with chronic aphasia who were evaluated two times each on two separate occasions (the test-retest PWA groups, see Table 1 in the Methods section), with no speech-language therapy in between. The ICCs (type A-1) for test-retest reliability for each of the subtests (calculated again using package irr [49]) are presented in Table 4. All repetition and most oral production subtests demonstrated excellent test-retest reliability according to the stringent criteria outlined by Koo and Li [48], with Verb Naming showing good and Discourse Production–moderate reliability. The lower reliability of the Discourse Production subtest, again, likely reflects the more subjective nature of the scoring for this subtest (as discussed in the previous section). Comprehension subtests showed variable reliability: more complex subtests revealed good (Lexical Decision, Sentence Comprehension, Discourse Comprehension) and moderate (Nonword Discrimination) reliability, while the single word comprehension subtests demonstrated poor reliability (Noun and Verb Comprehension). These low ICCs reflect high probability of responding at chance (i.e., limitations of a forced choice format), limited variance, and the ceiling effects observed in these subtests (cf., CAT [24], QAB [22]). If compared directly, the differences in scores between the two testing sessions for any of the RAT subtests were trivial and non-significant (paired-sample t-tests, *p* > .05). This observation in combination with good to excellent internal reliability estimates for all comprehension subtests implies that the below-normal scores on these subtests can still be confidently interpreted as such. Still, given that particularly single word comprehension subtests demonstrated poor test-reliability reliability, we would caution future test users against interpreting them in isolation or in contrast scores, especially for individual cases.

Also, since test-retest data was collected across two groups of PWA, we were not able to compute test-retest reliability of the GAQ. However, to get an estimate of the reliability of the overall test score, we computed a partial GAQ based on the available subtest scores in each of the two groups. ICCs of the GAQ-proxy were high in both instances supporting projected excellent reliability of the summary score. Taken together, the results of internal, inter-rater, and test-retest reliability analyses demonstrate that the RAT scores are highly stable across test administrations, with possible minor fluctuations in comprehension scores.

### Limitations

While the development and the introduction of the RAT into clinical practice presents a great advancement in comprehensive language assessment of Russian-speaking people with aphasia, the collected dataset has several limitations, which will need to be addressed by future investigations.

First of all, since technical errors precluded collection of test-retest data for all of the RAT subtests in a single sample, we could not establish test-retest reliability values for the overall test score–the GAQ. Since test-retest reliability of most of the RAT subtests is high along with high reliability of partial GAQ scores, we anticipate that overall test reliability will be excellent as well, however this still needs to be empirically demonstrated.

Next, more work is required to characterize the internal structure of the RAT. A much larger sample of PWA is needed to properly identify the test’s underlying factors and evaluate the internal structure of the subtests using specific estimators [47, 51].

Furthermore, we did not directly investigate sensitivity of the test to detecting a clinically meaningful change. Future studies that document behavioral changes with criterion-based tasks could inform the sensitivity of the test to detecting improvement (or decline) in language functioning and provide guidance to clinicians on how to interpret observed changes in the scores in a meaningful way. Regression-based approaches for determining clinically significant changes in the scores should also be explored in future work (e.g., [52]).

Another limitation of the current normative dataset is that we only collected data from individuals with brain damage who were diagnosed with aphasia. Future inquiries need to include persons with brain damage but without language impairments, so that sensitivity of the test specifically to language deficits rather than to general cognitive sequalae of brain injury (e.g., fatigue, compromised attention, memory, executive skills) is ascertained. While the selective relationships observed between different RAT subtests demonstrate that subtests are differentially sensitive to specific language deficits and underscore the test’s construct validity, data from a non-aphasic brain injury group would further raise the diagnostic value of the RAT.

Finally, the RAT currently does not assess reading and writing abilities. We hope that in the near future we will be able to develop and standardize these subtests as well.

## Conclusions

The RAT is the first comprehensive aphasia language battery in Russian, sensitive to a range of deficits and with properly established validity, inter-rater and test-retest reliability according to contemporary psychometric standards. It provides a multidimensional characterization of impaired and spared aspects of language functioning at different linguistic levels in different domains: auditory comprehension, repetition, and oral production. By using a ROC-curve analysis (which is rarely done in aphasia tests, with the QAB [22] being a notable exception), we optimized the test’s sensitivity and specificity to obtain excellent diagnostic characteristics across different age groups and aphasia types. Provided conversion of raw scores to severity ranks simplifies the test’s interpretation in clinical use, enabling meaningful comparison across subtests, patients, and time points. Notably, the RAT is the first comprehensive aphasia test in any language to be fully automatized for presentation on a tablet, maximizing further standardization of administration and scoring procedures, simplifying data collection, and facilitating record-keeping. The most recent tablet-based version of the RAT has identical functionalities in terms of stimuli presentation and response registration to the original version described in the Methods, but also has a user-friendly interface for scoring all the subtests on the device, comparing scores to normative data and reporting. This greatly facilitates clinical work, as it obviates the need for manual computation of scores, and clinicians can easily see how the evaluated patient compares to the normative sample in each of the subtests and to their own previous performance. We also hope that our carefully documented experience in designing and standardizing a tablet-based comprehensive aphasia test will serve both as an example and an inspiration for development of psychometrically sound and automatized aphasia batteries in other languages.

Overall, the results of the current standardization study clearly demonstrate that the RAT overall and its different subtests were differentially sensitive to language deficits in aphasia, and that the test is a valid and reliable tool for identifying language impairment in individuals with brain injury, quantifying aphasia severity, and providing a comprehensive evaluation of deficits at different processing levels across language domains. We hope that the tablet-based version of the RAT will be widely used in clinical and research settings, leading to substantial improvement in aphasia management for Russian-speaking patients.

## Supporting information

S1 File Detailed description of individual subtests of the RAT. (PDF)

S1 Table Descriptive statistics for accuracy subtest scores (% correct) of the RAT for the control group of neurologically healthy individuals (NHI) and the main group of people with aphasia (PWA) for each age cohort. (PDF)

S2 Table Cutoffs scores (%) and identified 34^th^ and 67^th^ percentiles for the PWA group used to determine aphasia severity ranks in each subtest and the General Aphasia Quotient (GAQ). (PDF)

S3 Table Item passing rates (item difficulty) of the RAT subtests based on the data of the PWA group. (PDF)

S4 Table Corrected item-total correlations of the RAT subtests based on the data of the PWA group. (PDF)
