# Supplementary material for: The Russian Aphasia Test: The first comprehensive, quantitative, standardized, and computerized aphasia language battery in Russian
Source: PLoS One. 2021 Nov 18;16(11):e0258946. doi: 10.1371/journal.pone.0258946 (PMC8601577; doi:10.1371/journal.pone.0258946)
Supplement: S1 File — (PDF) [file pone.0258946.s001.pdf]

S1 File. Detailed description of individual subtests of the RAT.

Auditory Comprehension Subtests

Nonword Discrimination

Discrimination of speech sounds is a common method for diagnosing deficits in comprehension of low-level auditory information, i.e., impairments at the acoustic-phonetic and/or phonological levels. The construction and the structure of this subtest was motivated by a similar subtest in the Psycholinguistic Assessment of Language Processing in Aphasia (PALPA; Kay et al., 2009), one of the few batteries that evaluates PWA's discrimination of nonword minimal pairs. Poor discrimination of nonwords indicates a deficit in acoustic-phonetic and/or phonological analysis.

The nonword discrimination subtest requires participants to listen to the auditorily presented pairs of nonwords (*n* = 22) and identify whether they are the same or different by touching the words 'yes' (same) or 'no' (different) on the tablet screen. Half of the pairs include the same nonwords, another half — different nonwords. Presentation of 'same' and 'different' pairs is pseudorandomized. The exact instructions for the task are formulated as follows: "You will hear pairs of syllables. Sometime syllables in the pair will be different, sometimes they will be repeated. Listen very carefully. Your task is to decide whether you hear the same syllables or not, and touch either "YES" or "NO". Touch "YES" if you think the syllables are the same. Touch "NO" if you think the syllables are different." Please note, that specifically for this project, we explicitly instructed both healthy controls and participants with aphasia to respond with the left hand, since we plan to carry out a reaction time analysis for the comprehension subtests and want to ensure that right-sided hemiparesis does not create a confound. However, for the general clinical use of the RAT, PWA are free to respond with whichever hand they prefer.

Only nonwords are used in the test in order to eliminate the lexical bias effect. Because PWA experience greater discrimination difficulties with consonants than vowels (Shankweiler & Harris, 1966; Trost & Canter, 1974), only consonantal distinctive features are manipulated in this subtest. Nonword pairs can be minimally different according to several phonetic features:

manner of articulation (/ro/-/lo/, *n* = 3), place of articulation (/bɛm/-/gɛm/, *n* = 3), palatalization (/rux/-/r^jux/, *n* = 3) or voicing quality (/druf/-/truf/, *n* = 2). Differences occur either in the word-initial (/pɛ/-/bɛ/, *n* = 8) or final (/ɛp/-/ɛk/, *n* = 3) positions. Due to consonantal devoicing in the word-final position in the Russian language, the voicing contrast occurs only in the stimuli's word-initial positions. Discrimination of nonwords based on the manner of articulation is expected to be easier than place of articulation or voicing quality (Gow & Caplan, 1996); data on discrimination of palatalization contrasts in PWA are lacking. All five Russian vowel phonemes (/i/, /ɛ/, /a/, /u/, /o/) are evenly distributed across the stimulus nonwords. Unlike in the PALPA battery, the syllabic structure of the stimuli is varied and includes the following patterns: VC (*n* = 6), CV (*n* = 4), CVC (*n* = 4), CCVC (*n*= 3), CVCC (*n* = 2), and CCVCC (*n* = 3). The correctly identified condition of an item (same or different) is scored as 1; otherwise, the item is scored as 0. Besides overall task performance, error analysis can highlight whether different distinctive features have differential impact on the veracity of acoustic-phonetic and phonological discrimination.

Lexical Decision

The auditory lexical decision task requires classifying spoken stimuli as real words or nonwords and involves both phonological and lexical levels of processing. Among existing diagnostic tools for aphasia, only the PALPA (Kay et al., 2009) uses an auditory lexical decision task, in which lexical frequency, imageability and morphological complexity of the lexical stimuli are varied. Higher error rates indicate impaired access to the phonological form of the word and/or a deficit in lexical processing (Goldinger, 1996).

The lexical decision subtest includes a total of 24 stimuli: 12 real words and 12 nonwords, presented auditorily in a pseudorandomized order. Participants are required to identify whether the presented linguistic items are real words or not. Participants touch the words 'yes' or 'no' on the tablet for real words and nonwords, correspondingly. The instructions for the task are: "You will hear real words and sound strings which sound like real words. Your task is to decide whether the sound that you hear is a real word or not, and touch either "YES" or "NO". Touch "YES" if you hear a real word. Sometimes the sound string will not make sense, in this case, touch "NO"."

Unlike in the PALPA, the current lexical decision subtest only uses highly imageable (*M* = 1.32, *SD* = 0.2) and low-frequency words (*M* = 3.05 ipm, *SD* = 2.15 ipm; the frequency values here

and further are taken from Lyashevskaya & Sharov, 2009). All words constitute nouns used in their citation form (nominative case, singular number). Nonwords are constructed from real words by changing one consonant sound in any position of the word, while retaining their phonotactic regularity, e.g., “дловарь” /dlɐˈvarʲ/ instead of “словарь” /slɐˈvarʲ/ (*a dictionary* → **mictionary*). Both real words and nonwords vary in length such that there are 12 2-syllable words and nonwords and 12 3-syllable items.

The correctly identified condition of an item is scored as 1; otherwise, the item is scored as 0.

Noun Comprehension & Verb Comprehension

Single word comprehension requires processing of incoming input at the phonological and lexical-semantic levels. Assessment of single word comprehension is usually included in most widely used language assessment batteries for aphasia, such as the Boston Diagnostic Aphasia Examination (BDAE; Goodglass, Kaplan & Barresi, 2001), the Western Aphasia Battery-Revised (WAB; Kertesz, 2007), the Comprehensive Aphasia Test (CAT; Swinburn et al., 2004), the PALPA (Kay et al., 2009), the Quick Aphasia Battery (QAB; Wilson et al., 2018). Word-to-picture matching is the most common task, although there is little consistency in the types of manipulated variables in the auditory and visual stimuli. This task is used for assessing word level comprehension abilities, and the level of breakdown can be additionally established, depending on the task design. Currently, only comprehension of nouns is tested in comprehensive aphasia batteries (but see Assessment of Speech in Aphasia, ASA; Tsevtkova et al., 1981). Yet, recent research demonstrates that comprehension of verbs is more challenging than comprehension of nouns and, therefore, should be thoroughly assessed as well (Soloukhina & Ivanova, 2017).

The single word comprehension subtest examines comprehension of both nouns (n = 24) and verbs (n = 24), which are presented to participants in two separate subtests. Each word is presented auditorily together with four pictures: of the target word and the phonological, semantic, and unrelated distractors (similar to the CAT comprehension subtest; Swinburn et al., 2004; see Figure A1 for examples). The task is to match the target word to its corresponding picture. The position of the target pictures on the screen is balanced. The instructions for the noun comprehension and the verb comprehension subtests are the same: “You will hear a word

and will see four pictures. One of the pictures corresponds to the word, while the remaining three - do not. Touch the picture that corresponds to the word.”

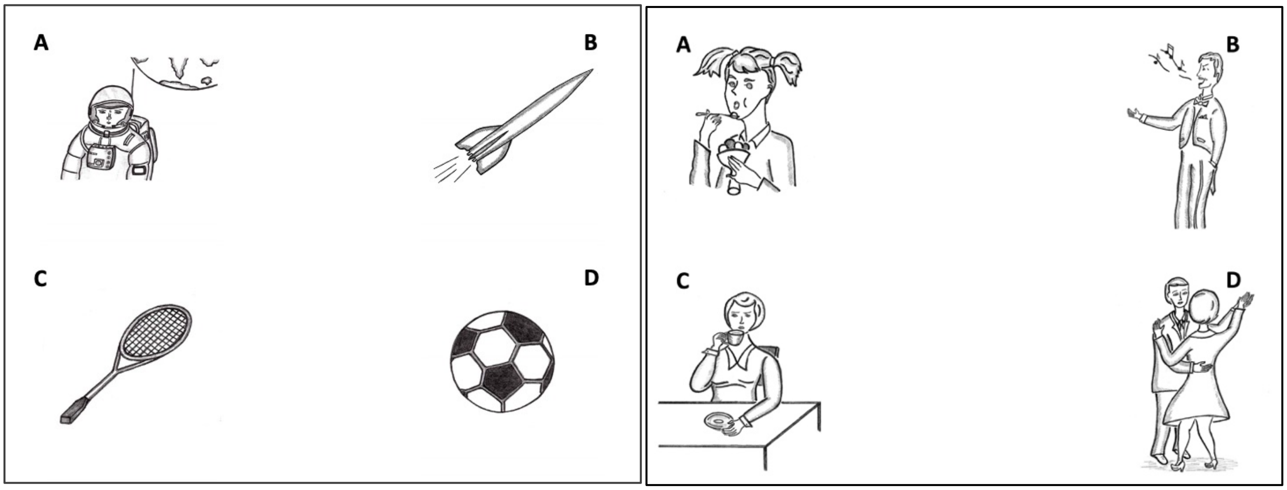

Figure A1. Example trials from the single word comprehension subtests. Left panel – noun comprehension subtest: target word “ракета” (*a rocket*) (B); a semantically related distractor, “космонавт” (*an astronaut*) (A); a phonological distractor, “ракетка” (*a racket*) (C); an unrelated distractor, “мяч” (*a ball*) (D). Right panel – verb comprehension subtest: target word “петь” (*to sing*) (B); a semantically related distractor, “танцевать” (*to dance*) (D); a phonological distractor, “пить” (*to drink*) (C); an unrelated distractor, “есть” (*to eat*) (A). Letters are only provided in this figure for illustration purposes.

Phonological distractors differ from the target word by 1-2 phonemes in any position of the word, e.g., “корона” /kɐˈronə/ (*a crown*) – “корова” /kɐˈrovə/ (*a cow*), “сушить” /sʊʂˈɨtʲ/ (to dry) – “душить” /dʊʂˈɨtʲ/ (*to choke*). Semantic distractors are related to the target taxonomically or thematically, e.g., “петь” (*to sing*) – “танцевать” (*to dance*); “ракета” (*a rocket*) – “космонавт” (*an astronaut*). The unrelated foils have no phonological or semantic relation to the target, although they are semantically related to the phonological distractors in order to minimize the use of metalinguistic strategies. The relevant psycholinguistic parameters for the stimulus words (age of acquisition, imageability, frequency, length in syllables) and pictures (name agreement, subjective and objective complexity, familiarity) are balanced between the nouns and verbs (Table A1). This ensures that observed differences between the two subtests, if any, pertain to grammatical class processing discrepancies. Additionally, target words and pictures in this subtest are matched with the stimuli in the naming subtest, allowing for comparison of PWA’s performance across comprehension and production tasks (Table A1).

A correct match between the word and the picture is scored as 1; otherwise, the item is scored as 0. Error analysis can indicate whether difficulties in word comprehension stem from breakdowns at the phonological and/or the lexical-semantic levels.

Table A1. Psycholinguistic parameters for target words and pictures in the single word comprehension and naming subtests.

Subtest	Word-class	Picture-related parameters, mean (SD)			Word-related parameters, mean (SD)				
		Name agreement, %	Subjective visual complexity	Familiarity	Age of acquisition	Imageability	Image agreement	Frequency, ipm	Length, syllables
Single word comprehension	Noun	93.8 (5.4)	2.7 (0.5)	3.9 (0.6)	1.8 (0.5)	1.1 (0.1)	4.2 (0.4)	29.3 (26.8)	2.2 (0.7)
	Verb	90.3 (7.6)	2.5 (0.4)	3.9 (0.7)	1.7 (0.4)	1.2 (0.1)	4.1 (0.7)	46.4 (79.9)	2.7 (1.0)
Naming	Object	93.5 (4.7)	2.6 (0.5)	4.1 (0.6)	1.9 (0.5)	1.1 (0.1)	4.4 (0.2)	28.4 (31.3)	2.4 (0.7)
	Action	91 (8.4)	2.5 (0.4)	3.7 (0.7))	1.8 (0.4)	1.2 (0.2)	4.3 (0.5)	33.9 (47.2)	2.5 (0.8)

Note. *Name agreement*: proportion of the most frequently elicited name to all responses in the normative naming task; *Subjective visual complexity*: picture complexity, rated on a scale from 1 (simple) to 5 (complex); *Familiarity*: familiarity of an object/action, rated on a scale from 1 (barely familiar) to 5 (very familiar); *Age of acquisition*: rated age of word acquisition on an interval scale from 1 (0-3 years) to 5 (later than 12 years), with three-year intervals in between; *Imageability*: effort required to imagine an object/action, rated on a scale from 1 (easy to imagine) to 5 (difficult to imagine); *Image agreement*: agreement of the picture with the mental image evoked by the word, rated on a scale form 1 (no agreement) to 5 (excellent agreement); *Frequency*: lemma frequency according to (Lyashevskaya & Sharoff, 2009). See (Akinina et al., 2014, 2015, 2016), for further details on the normative studies.

Sentence Comprehension

Comprehension at the sentence level requires morphosyntactic processing in addition to phonological and lexical-semantic processing. Unlike comprehension of isolated spoken words, sentence-level comprehension requires the listener to identify relationships among the words by means of word order cues, grammatical markers, prepositions, and semantic knowledge, in order

to be able to extract the sentence meaning (e.g., Caramazza & Berndt, 1978; Schumacher et al., 2015). Typically, sentence comprehension in aphasia is either tested with a sentence-to-picture matching task (e.g., CAT, Swinburn et al., 2004) or execution of commands (e.g., WAB, Kertesz, 2007; Token Test, de Renzi & Flaglioni, 1978).

To assess PWA's sentence-level auditory comprehension ability in the RAT, we use a sentence-to-picture matching task. The advantage of this task, compared to execution of commands, is that the latter requires a fine motor response and therefore might be compromised in some individuals post stroke. Each trial (n = 24) contains a sentence presented auditorily, and the participants are asked to choose one of the two pictures presented on the screen. The instructions for the subtest are as follows: "You will now hear a sentence and see two pictures. One of the pictures corresponds to the sentence, the other one does not. Touch the picture that corresponds to the sentence." The sentence is presented in a form of a question which sounds more naturally in the context of the task but does not change the structure of the sentence after the question word. One of the pictures (the target) corresponds to the sentence; the other one serves as a distractor (see Figure A2, for an example). The positions of the target and distractor pictures on the screen are balanced and pseudorandomized across trials.

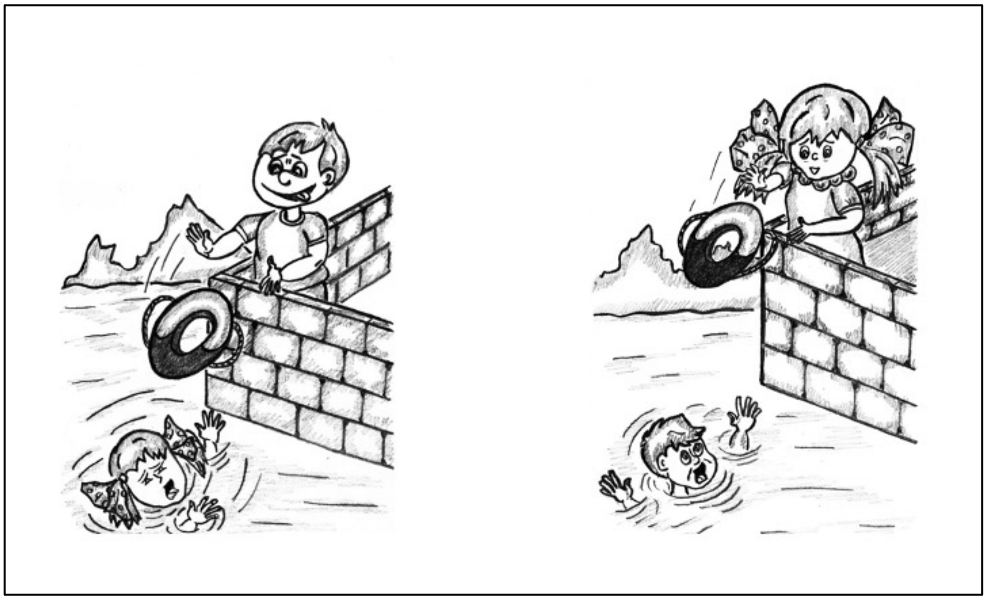

Figure A2. An example of an item in the sentence comprehension subtest. The stimulus sentence is "Где мальчик спасает девочку?" where boy.NOM save.PRS.3 girl.ACC (*Where is the*

boy saving the girl?). The target picture is on the left; the distractor picture (corresponding to a reversed situation where the girl is saving the boy) is on the right.

The stimuli contain constructions of various complexity and include sentences with:

1) simple syntactic structures (*n* = 9), e.g., "Где мальчик спасает девочку?" where boy.NOM save.PRS.3 girl.ACC (*Where is the boy saving the girl?*);
2) subject and object relative clauses (*n* = 9), e.g., "Где мальчик, который ловит бабочку?" where boy.NOM who.NOM catch.PRS.3 butterfly.ACC (*Where is the boy who is catching the butterfly?*) / "Где бабуля, которую кормит внучка?" where grandmother.NOM who.ACC feed.PRS.3 granddaughter.NOM (*Where is the grandmother who is being fed by the granddaughter?*);
3) prepositional constructions (*n* = 6), e.g., "Где мальчик кладет в сумку колбасу?" where boy.NOM put.PRS.3 in bag.ACC sausage.ACC (*Where is the boy putting the sausage in the bag?*).

In each of these constructions (except the two SV sentences), word order is varied such that half of the sentences have a canonical (unmarked) SVO word order in Russian (Baylin, 2011) and the other half have a non-canonical (marked) OVS word order, e.g., where boy.NOM save.PRS.3 girl.ACC (SVO; *Where is the boy saving the girl?*) vs. where girl.ACC frighten.PRS.3 boy.NOM (OVS; corresponds to the English passive construction – *Where is the girl being frightened by the boy?*). For the prepositional constructions, the canonical word order is the direct object followed by the prepositional phrase for arguments of ditransitive verbs (DP > PP), while the opposite pattern marks the non-canonical order (Dyakonova, 2009). Additionally, 16 sentences are semantically reversible: the reversed interpretation of syntactic roles of the constituents results in a semantically plausible sentence, e.g., *Where is the boy saving the girl?* vs. *Where is the girl saving the boy?*. The distractor pictures for such sentences always depict a semantically reversed situation (see Figure A2, for an example). The remaining 8 sentences are semantically irreversible, e.g., *Where is the old woman knitting a sweater?* (the semantically reversed counterpart is not semantically plausible: **Where is the sweater knitting an old woman?*). The distractor pictures for irreversible constructions depict a different action with the same subject-object pair, e.g., an old woman knitting (target) / washing (distractor) a sweater,

where the paired verbs are matched for lexical frequency. Similarly, for the two SV sentences a distractor depicts the same subject performing a different action. Sentences are presented in the order theoretically representing increasing syntactic complexity: simple syntactic structures < sentences with relative clauses < prepositional constructions. Sentences within each group are pseudorandomized.

A correct choice of the target picture is scored as 1; otherwise, the item is scored as 0. Analysis of errors across different items allows to determine the extent and severity of syntactic deficits. Also, syntactic constructions used in this subtest are matched with the stimuli in the sentence production subtest, allowing for comparison of PWA's performance between comprehension and production tasks.

Discourse Comprehension

Successful comprehension of spoken language, in addition to phonological, lexical-semantic and syntactic processing, also requires pragmatic skills, such as constructing inferences and understanding the connections between the elements of discourse (see Carpenter, 1995; Nicholas & Brookshire, 1995, for a review). It also puts a heavier burden on working memory, making discourse comprehension one of the hardest tasks for PWA. Nevertheless, evaluating discourse comprehension is important due to its ecological validity, since comprehension of the narratives is a fundamental part of human communication (Mar, 2004). Tasks assessing spoken or written discourse comprehension are included in many aphasia batteries (e.g., CAT, Swinburn et al., 2004; BDAE, Goodglass, Kaplan & Barresi, 2001). A typical spoken discourse comprehension task implies oral presentation of brief stories followed by yes/no questions.

To assess discourse comprehension in the RAT, we constructed an original narrative, The Cat Story, and 16 follow-up statements. Participants listen to the story in its entirety and verify if the follow-up statements are true or false. The following exact instructions are given to the participants: "You will hear a story. Listen carefully and try to remember it. Afterwards, you will hear statements about the events in the story. Your task is to decide wither they are true or false, and touch either "YES" or "NO". Touch "YES" if you think the statement describes the story accurately. If not, touch "NO"." The statements, presented in a pseudorandomized order, are intrinsically paired; each pair is related to one fact mentioned in the story, so that one statement

is true and the other is false. PWA can rely on heuristic processes and their knowledge about the world when performing discourse comprehension tasks (e.g., see Kintsch & van Dijk, 1978; van Dijk & Kintsch, 1983), which can potentially result in scores that are not directly related to sentence-level comprehension scores (Caplan & Evans, 1990; Ferstl et al., 2005). To minimize this, The Cat Story does not resemble any well-known plots (e.g., a fable, a tale or a movie). The plot is the following. A couple (Natasha and Dima) goes on a picnic to the lake. They take a pie made by Natasha with them. Dima carries the heavy backpack with the pie and wonders why it is so heavy. When Natasha and Dima arrive at the place of the picnic and open the backpack, they find their cat sleeping there. The story is 156 words long (mean content word frequency = 261.32 ipm; median = 94.7 ipm) and contains 31 clauses (clause length in words: M = 5.03, *min* = 2, *max* = 10), all in canonical word order; 4 clauses are relative object clauses.

The characteristics of the follow-up statement pairs - salience and explicitness of the information - are motivated by the Discourse Comprehension Test (DCT, Brookshire & Nicholas, 1993). Each of the statement pairs corresponds to an event from the main plot or a detail (salience) and pertains to either explicit or implicit information (explicitness). For example, the fact that Natasha made a pie for the picnic is important for understanding of the plot, while the fact that the picnic took place under a big tree only adds an additional detail. The explicit information is stated in the text (e.g., the fact that Natasha and Dima went on a picnic), while the implicit information requires constructing inferences and pragmatic knowledge (e.g., there is no direct mention of the fact that the cat had gotten into the backpack at home). The 8 pairs of statements are arranged in a 2 x 2 design, which allows to differentially evaluate the impact of these different parameters on comprehension accuracy.

A point is given for a statement pair if the participant correctly evaluates both statements; thus, the maximum total score for this subtest is 8. The impact of the two statement parameters (salience and explicitness) can be differentially evaluated.

Repetition Subtests

Nonword Repetition

The nonword repetition task taps into many language processes, including speech perception, phonological encoding, phonological working memory, phonological planning, assembly and articulation (Coady & Evans, 2008; Gathercole et al., 1994; Gathercole, 2006). Impaired nonword repetition may indicate deficits in any or several of these processes. This task is commonly used in many comprehensive batteries, such as the BDAE (Goodglass, Kaplan & Barresi, 2001), the CAT (Swinburn et al., 2004), and the PALPA (Kay et al., 2009).

In the present subtest, participants listen to 24 nonwords presented auditorily in a pseudorandomized order and repeat them. The instructions are as follows: "You will hear sound strings that sound very similar to real words. Repeat them after the speaker as precisely as you can. You will hear each sound string once, so listen very carefully. Listen to the whole sound string and repeat it."

The wordlikeness of the nonwords was manipulated such that half of the nonwords (n = 12) were created by replacing 1 phoneme in a real Russian word in any position of the word (high wordlikeness condition), while the other half (n = 12) were constructed by transposing the syllables while still preserving the Russian phonotactic regularities (low wordlikeness condition). Both types of nonwords are balanced in terms of length: there is an equal number of 1-, 3- and 5-syllable nonwords. A wordlikeness effect on nonword repetition and its interaction with the nonword length (less accurate performance on low-wordlike than high-wordlike nonwords, especially for longer nonwords) may indicate difficulty in maintaining stable phonological representations and impairment of the output buffer (Estes et al., 2007; Saito et al., 2003).

Additionally, the stimuli vary in articulatory complexity, which is defined as a number of transitions between primary place of articulation of any two adjacent consonants, i.e., articulatory switches, within a word. E.g., "дот" /dot/ has 0 articulatory switches because both consonants in the word are coronal consonants (they have the same place of articulation), while "тап" /tap/ has 1 articulatory switch because /t/ is a coronal consonant and /p/ is a labial consonant. The nonwords in this subtest have between 0 and 5 articulatory switches. A positive relationship between the number of articulatory switches and the error rate (the more switches, the more errors) may indicate disturbance of the programming and execution of speech movements characteristic of apraxic speech (Deger & Ziegler, 2002).

Correct repetitions of nonwords are scored as 1, phonological paraphasias (more than 50% of the target is spared, and the target is still recognizable) are scored as 0.5, any other repetition errors or no response are scored as 0. The effects of the wordlikeness, length and articulatory complexity can be additionally examined.

Word Repetition

The word repetition task targets the same linguistic processes as the nonword repetition task, but also involves the lexical-semantic level. Comparing PWA's performance on nonword vs. word repetition may reveal lexical effects in word processing and contribute to the understanding of where the repetition difficulties stem from. For example, unstable phonological representation hypothesis (Saito et al., 2003; Yamadori & Ikumura, 1975) predicts a lexicality effect, with nonwords posing a greater difficulty for repetition than real words, because impairment of phonological output buffer typically affects nonwords more than real words. On the contrary, absence of the lexicality effect may suggest post-lexical difficulties, e.g., in the execution of the motor plan, which may affect repetition of words and nonwords similarly. Word repetition tasks are routinely included in comprehensive aphasia batteries, such as the WAB (Kertesz, 2007), BDAE (Goodglass, Kaplan & Barresi, 2001), CAT (Swinburn et al., 2004), and PALPA (Kay et al., 2009). The present subtest is similar to the corresponding subtests in the CAT (Swinburn et al., 2004) and the PALPA (Kay et al., 2009), as it manipulates word length and word frequency. But unlike the existing tasks, the RAT version of the task additionally takes into account articulatory complexity.

The subtest includes 24 existing Russian words presented pseudorandomly which participants are asked to repeat. The exact instructions are as follows: "You will hear words. Your task is to repeat them after the speaker as precisely as you can. You will hear each word once, so listen very carefully. Listen to the word and repeat it." There is an equal number of 1-, 3- and 5-syllable words in the subtest. Half of the words in each length group have high lemma frequency (*M* = 220.74 ipm, *SD* = 92.81 ipm), and the other half have low lemma frequency (*M* = 3.95 ipm, *SD* = 2.38 ipm). PWA with deficits at the lexical level are expected to experience more difficulty with low-frequency compared to high-frequency words (Haley & Jacks, 2014; Nozari et al., 2010). Additionally, to provide a direct comparison with the nonword repetition task, articulatory

complexity of the words (a total number of articulatory switches; see description of the nonword repetition subtest above for an explanation) is also varied and has a similar range (from 0 to 5 articulatory switches).

Correct word repetitions are scored as 1, phonological paraphasias are scored as 0.5, any other repetition errors or no response are scored as 0. The effects of lexical frequency, length and articulatory complexity can be additionally evaluated.

Sentence Repetition

Beyond targeting processes evaluated by the word repetition task described above, the sentence repetition task allows to evaluate auditory short-term verbal memory deficits, by incorporating sentences of varying length. Sentence repetition is typically included in aphasia batteries, although different parameters of sentences are rarely systematically manipulated. For instance, the CAT (Swinburn et al., 2004), PALPA (Kay et al., 2009), and BDAE (Goodglass et al., 2001) manipulate sentences' length but not lexical frequency, while it is a critical parameter of lexical access. Lexical frequency of the sentences' content words is manipulated in the WAB (Kertesz, 2007), however, it is not separated from the length factor, and repetition of words is not scored separately from repetition of sentences.

In the current sentence repetition subtest, participants listen to sentences (n = 12) and repeat them. They receive the following instructions: "You will now hear a sentence. Your task is to repeat it after the speaker. Listen to each sentence carefully and repeat it." The length of the sentences (3-4 words vs. 6-9 words) and the lexical frequency (high: M = 728.93 ipm, SD = 369.94 ipm vs. low: M = 9.84 ipm, SD = 4.46 ipm) of content words are systematically varied in a crossed design, with 3 sentences per each condition. The length effect (greater difficulty with repeating longer sentences) indicates auditory verbal short-term memory deficits. The difference between repetition of sentences with high- and low-frequency lexical items is critical for characterizing both lexical access impairments and memory deficits, since serial repetition of low frequency items is more challenging (Mulligan, 2001). The 2 x 2 design allows to evaluate independent and cumulative impact of these factors. The hardest condition of the current subtest – long sentences with low-frequency items – helps to detect even mild repetition deficits.

The scoring of the subtest is done at the word level. For each correctly repeated word (including functional words) in the sentence, 1 point is given. Phonological paraphasias and word form errors are scored as 0.5, while omissions and other errors are given a score of 0. Word order changes, omissions and insertions (altering the overall order), repetitions or word searches incur an order penalty of 1 (irrespective of the number of such errors). The score for each sentence is calculated as the sum of points minus the order penalty. E.g., for the target sentence "*The capricious baroness criticizes the intricate floral ornament*", the response "*The capricious baroness criticize the capricious ornament*" will be evaluated as the following: *the capricious* (1, correct) + *baroness* (1, correct) + *criticize* (0.5, wrong form) + *the capricious* (0, perseveration) + *ornament* (1, correct) -1 for the word omission; resulting in a score of 2.5 for this item. The total score for the subtest is the sum of scores for all the items. Additionally, the effects of length and lexical frequency can be separately examined.

Oral Production Subtests

Object Naming & Action Naming

Object naming represents a gold standard in assessment of the language function because it necessitates multiple cognitive computations, such as visual object recognition, semantic-conceptual processing, lexical access, phonological encoding, speech planning and articulation (Whitworth, Webster & Howard, 2005). Due to its versatility and the fact that anomia is the most ubiquitous deficit in aphasia, object naming is widely used in the majority of aphasia test batteries. Additionally, a body of neurolinguistics evidence suggests that object and action naming may dissociate in PWA and probably rely on only partly overlapping neural circuits (e.g., Mätzig et al., 2009; Vigliocco, Vinson, Druks, Barber, & Cappa, 2011). There are several tests, such as the Verb and Sentence Test (VAST; Bastiaanse, Maas, & Rispens, 2000), the Object and Action Naming Battery (Druks & Masterson, 2000), and the Northwestern Naming Battery (NNB; Thompson et al., 2012) that have been developed recently to specifically provide additional assessment of action naming in PWA. Still, action naming is typically not included in traditional aphasia batteries with the exception of the ASA (Tsevtkova et al., 1981), extended version of the

BDAE (Goodglass, Kaplan & Barresi, 2001) and recently the CAT (Swinburn et al., 2004), although the latter two use an unbalanced design.

To address this problem and provide a more balanced solution, the RAT includes naming of both objects (*n* = 24) and actions (*n* = 24), which are presented to participants in two separate subtests, one picture at a time. Participants have to name the presented picture with one word. In the object naming subtest, participants are given the following instructions: "You will see a picture. Look at it carefully and say what is depicted in the picture with one word." In the action naming subtest, participants are told: "You will see a picture. Look at it carefully and say what the character or the characters in the picture are doing with one word." Similar to the noun and verb comprehension subtests, the object- and action-related stimuli are matched on the same psycholinguistic parameters (pictures: name agreement, subjective complexity, familiarity; words: age of acquisition, imageability, frequency, length in syllables; see Table A1 for specific values). The values are also matched to the stimuli in the single word comprehension subtest, thereby enabling direct comparison of participants' scores not only between object and action naming, but also between naming and comprehension.

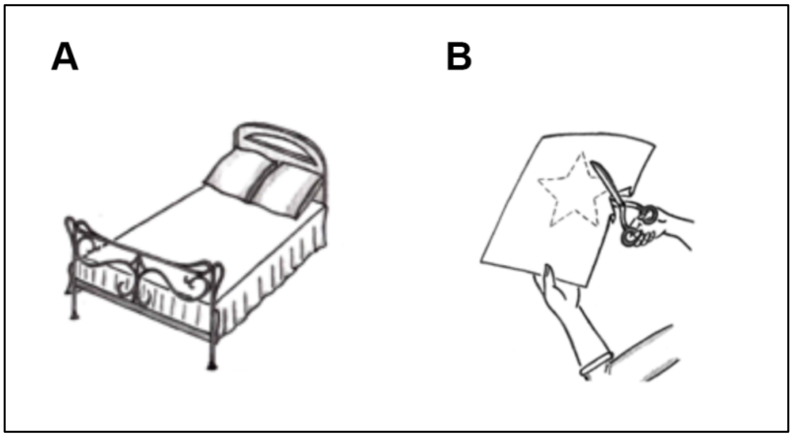

Figure A3. An example of a trial in the naming subtests. A) Object naming subtest (target noun — "кровать" (*a bed*)), B) action naming subtest (target verb — "вырезать" (*to cut out*)).

Correct naming, defined as elicitation of the target word or one of the acceptable alternatives, is scored as 1. List of acceptable alternatives for each item is based on responses that occurred in at least 2% of norms in the Database of Russian Verbs and Nouns (Akinina et al., 2014, 2015, 2016). Different morphological word forms (e.g., plural and singular forms for nouns,

infinitive or tense forms for verbs, etc.), diminutive forms and the use of the target word within a phrase (e.g., “reads a book” instead of “read”) are permitted. Word searches resulting in correct naming and dysarthric speech (i.e., effortful or slurred articulation) are not penalized. Erroneous responses (semantic, phonological and mixed paraphasias, neologism, circumlocution, perseveration, onomatopoeia, wrong part of speech, etc.) or absence of response are all scored as 0. Additional analysis of error types and effects of psycholinguistic parameters on the responses can be performed to determine the locus of the impairment.

Sentence Production

The sentence production task assesses the ability to generate well-formed language output at multiple levels. In addition to processes targeted by the naming tasks, it also involves morphosyntactic processing. While standardized aphasia batteries typically evaluate sentence production, among which are the BDAE (Goodglass, Kaplan & Barresi, 2001), WAB (Kertesz, 1982), Northwestern Assessment of Verbs and Sentences (NAVS; Cho-Reyes & Thompson, 2012), and VAST (Bastiaanse, Maas, & Rispens, 2000), the nature of the tasks and the types of examined syntactic constructions vary greatly across different tests. The present subtest partly emulates the procedure of the NAVS’s subtest called Sentence production priming test (Cho-Reyes & Thompson, 2012). The crucial difference between the two tests is that, in our version, the priming and the primed stimuli differ in the actions they depict and the choice of the lexical items they necessitate.

Participants are presented with picture pairs (*n* = 24) and receive the following instructions: “You will now see two pictures and will hear an example sentence describing the picture on the left. Following this example, your task is to construct a sentence that would describe the picture on the right in a similar way.” The picture on the left, presented along with a sentence using a specific syntactic construction, is used to prime, or model, the participant’s response, while the picture on the right is used to elicit the participant’s response. For example, a picture of a bride and a groom riding in a car is accompanied by the priming sentence: “Невесту везет жених” bride.ACC give ride.PRS.3 groom.NOM (corresponds to the English passive construction *The bride is given a ride by the groom*; see Figure A4). The target picture on the right depicts a girl feeding an old man, and the participant is prompted to describe the situation

using the same syntactic construction as in the priming sentence: “Дедушку кормит девочка” grandfather.ACC feed.PRS.3 girl.NOM (corresponds to the English passive construction *The grandfather is fed by the girl*).

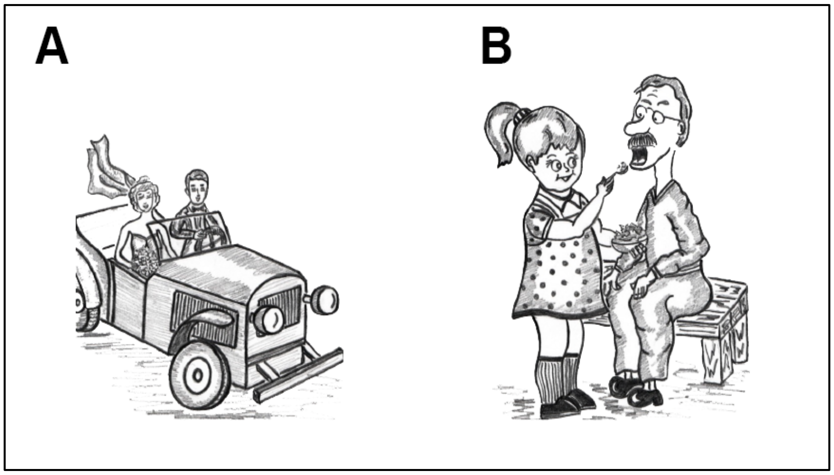

Figure A4. An example of a trial in the sentence production subtest. A) A model picture accompanying a priming sentence, “Невесту везет жених” bride.ACC give ride.PRS.3 groom.NOM (*The bride is given a ride by the groom*), B) A picture for the elicitation of the target response, “Дедушку кормит девочка” grandfather.ACC feed.PRS.3 girl.NOM (*The grandfather is fed by the girl*).

Intended target sentences range in syntactic complexity and include the same syntactic constructions as stimuli in the subtest on sentence comprehension, thereby allowing for a direct comparison of PWA’s performance on sentence comprehension and production. Accordingly, the target sentences include:

1) simple syntactic structures (*n* = 9), e.g., “Девочка шьет юбку” girl.NOM sew.PRS.3 skirt.ACC (*The girl is sewing a skirt*);
2) subject and object relative clauses (*n* = 9), e.g., “Девочка, которая рисует птичку” girl.NOM who.NOM paint.PRS.3 bird.ACC (*The girl who is painting the bird*) /”Косточка, которую нюхает собака” bone.NOM which.ACC smell.PRS.3 dog.NOM (*The bone that is smelled by the dog*);
3) prepositional constructions (*n* = 6), e.g., “Девочка кладет бочку в коробку” girl.NOM put.PRS.3 barrel.ACC in box.ACC (*The girl is putting the barrel in the box*).

In each of these sentence types, similarly to the sentence comprehension subtest, word order can be either canonical (SVO and two SV sentences; *n* = 13) or non-canonical (OVS; *n* = 11); sentences can be either semantically irreversible (*n* = 8) or reversible (*n* = 16). All sentences contain high-frequency lexical items that do not repeat between priming and (intended) target sentences to exclude a possibility of rote repetition. With the exception of two SV sentences, transitive verbs are used in the sentences. All the primes are prerecorded and presented automatically together with the visual stimuli. Similar to the sentence comprehension subtest, items are presented in the order loosely representing increasing syntactic complexity: simple syntactic structures < sentences with relative clauses < prepositional constructions. Sentences within each group are pseudorandomized.

Each response is scored based on the fulfilment of the four criteria: consistency with the prime (whether the required syntactic construction is used in the response), grammaticality (whether the sentence is grammatically correct, including correct use of sentence constituents, morphological marking, and thematic role assignment, irrespective of the conformity to the prime), lexical-semantic adequacy (with respect to the provided picture), and other aspects of phrase appropriateness (absence of excessive word finding difficulties, circumlocutions, false starts, repetitions, or metacommentary that impede intelligibility). A score of 1 is given for each criterion if it is met, and 0 if it is not. The sum of the scores across the four criteria constitute the score for a particular item. An item receives a score of 0 if no response is provided, if the response is completely irrelevant, if the verb is missing, or if neither of the four criteria is met. Analysis of individual criteria and error patterns across different items allows to determine the level of breakdown in sentence production and the severity of syntactic deficits. Also, syntactic constructions used in this subtest are matched to the ones in the sentence comprehension subtest, allowing for comparison between comprehension and production.

Discourse Production

Spoken discourse provides valuable information for the assessment of impairments in production at various linguistic levels similar to sentence production and also implicates a discursive-pragmatic component (see Bryant, Ferguson & Spencer, 2016, for a review). Typically assessed speech samples include spontaneous speech from conversations (e.g., BDAE,

Goodglass, Kaplan & Barresi, 2001; QAB, Wilson, Eriksson, Schneck & Lucanie, 2018) or picture-elicited discourse (e.g., BDAE, Goodglass, Kaplan & Barresi, 2001; CAT, Swinburn, Porter & Howard, 2004).

In the RAT, picture-elicited spoken discourse is assessed using a single stimulus picture, A Biking Mishap. The instructions and the picture are designed to motivate participants to produce a narrative rather than a description. Unlike descriptions, narratives have a more fixed discourse structure with essential elements arranged in a sequence and connected to each other. Thus, while both discourse types can be used for measuring microlinguistic and some macrolinguistic parameters (for review see Armstrong, 2000; Pritchard, Hilari, Cocks & Dipper, 2017), narrative analysis allows to assess the ability of the speaker to maintain the discourse structure and the order of events. Typical clinical elicitation instructions for the picture descriptions are ''Tell me what you think is happening in this picture''. However, Olness (2006) demonstrated that with such instructions, participants tend to produce descriptive discourse rather than narratives, and with instructions that directly request temporal sequencing, participants produce more narratives. Based on these guidelines, the instructions for the discourse production subtest are formulated as follows: "Look at the picture and tell me a story with a beginning, a middle part and an ending. From your story, it should be clear what happened at the beginning, what happened next and how it all ended. Let me know when you are ready to start." The 'beginning', the 'middle part' and the 'ending' in the instructions correspond to the exposition, climax and resolution of the narrative. The picture depicts three human characters and two animals: a girl riding a bike, a little girl running, an old man dropping a basket with apples, and a dog chasing a cat (see Figure A5). The picture allows several interpretations of causal connections between the characters and resulting events (e.g., the old man has dropped the basket because of the girl on the bike or because of the running animals), thereby eliciting richer, more complex and varied discourse samples.

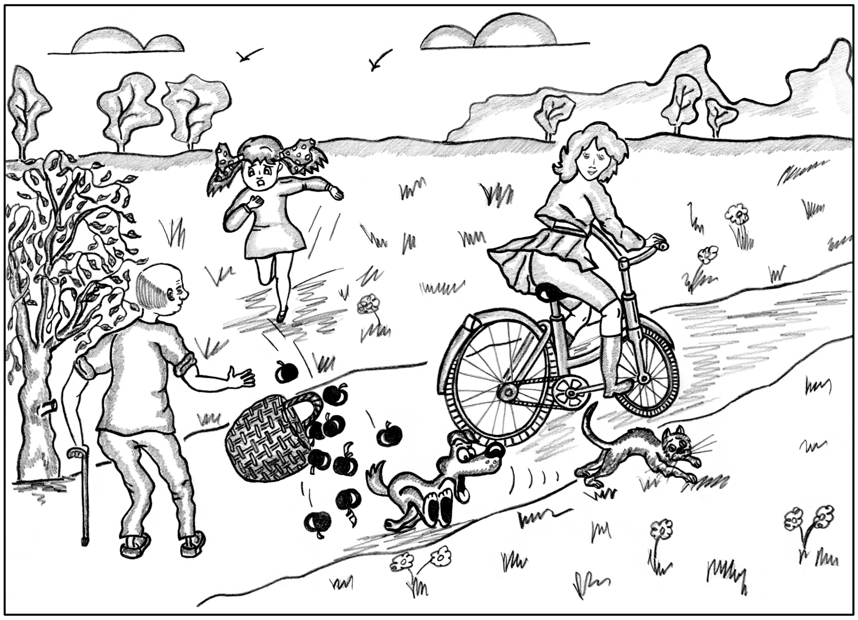

Figure A5. The stimulus picture (A Biking Mishap) used in the discourse production subtest.

Produced discourse is rated from 1 (severely impaired) to 5 (no impairment) on four scales reflecting distinct aspects of connected speech: fluency, grammatical complexity, amount of paraphasias, and information content (see Table A2, for a detailed description). Using several scales allows to assess discourse production at different language levels, both micro- and macrolinguistic, cf. similar approaches in the Aachen Aphasia Test (Huber, Poeck & Willmes, 1984), BDAE (Goodglass, Kaplan & Barresi, 2001) and QAB (Wilson et al., 2018), while remaining relatively easy and minimally time-consuming for the examiner (Prins & Bastiaanse, 2004). If the participant is unable to produce 5 content words related to the picture in one minute, they get a 0 score on all the scales. The total score of the subtest is the sum of scores on all four scales.

Table A2. Detailed description of rating scales used in the discourse production subtest.

Score / Scale	1	2	3	4	5
**Fluency**	Spontaneous speech is almost absent. Speech consists of automatisms; single content words might be present; there are many pauses.	Speech is non-fluent and dominated by isolated words; there are many pauses, perseverations and false starts.	Speech is non-fluent and/or telegraphic. Speech is dominated by isolated words and short phrases or sentence fragments; there are many pauses.	Speech is relatively fluent. There are pauses resulting from word-finding and/or phrase-forming difficulties.	Speech is fluent. There are no pauses resulting from word-finding or phrase-forming difficulties.
**Grammatical complexity**	Speech is characterized by severe agrammatism. Speech consists mostly of isolated words that are not organized into sentences.	Speech is agrammatic. Speech consists of isolated words and/or simple phrases, often with omissions of principal sentence constituents. Several simple nuclear phrases are present, possibly with agrammatical agreement.	Speech is agrammatic. Mostly simple sentences are used with frequent agreement errors. Some sentence constituents and functional words are omitted.	Speech is mostly grammatical, there are occasional agreement errors, some functional words might be omitted. Speech consists mostly of simple sentences. Attempts at using complex syntactic structures (participial phrases, relative clauses etc.) are made.	Speech is grammatical. There are complex sentences consisting of two or more independent or dependent clauses. There are no agreement errors.

Scale \ Score	1	2	3	4	5
**Paraphasias**	Frequent verbal and phonological paraphasias, neologisms and semantically empty words without corrections. The number of mistakes is greater than the number of correct content words.	There are frequent verbal and phonological paraphasias that affect the meaning of the phrase, and semantically empty words.	Verbal and phonological paraphasias are present, but do not affect the meaning of the phrase significantly. Semantically empty words occur periodically, sometimes with corrections.	There are occasional paraphasias, semantically empty words and/or occasional word-finding difficulties.	There are no paraphasias.
**Information content**	The story is uninformative. Some of the plot elements are present in an unorganized and incoherent manner and/or some of the characters are listed.	The informativeness of the story is low. Some of the plot elements are described, and an outline of a coherent story is present.	All main plot elements are present, but they are not connected and/or there are logical mistakes in the narration.	The story is informative. The main storyline is present without additional details.	The story fully corresponds to the events in the picture: all main plot elements are present and arranged logically, and there are some additional details.

References

Akinina, Y., Malyutina, S., Ivanova, M., Iskra, E., Mannova, E., & Dragoy, O. (2015). Russian normative data for 375 action pictures and verbs. *Behavior Research Methods, 47*(3), 691–707.

Akinina, Yu., Grabovskaya, M., Vechkaeva, A., Ignatyev, G., Isaev, D., & Khanova, A. (2016). Biblioteka psiholingvisticheskih stimulov: novye dannye dlja russkogo i tatarskogo jazyka [Psycholinguistic stimulus database: new data for Russian and Tatar]. In Y. Alexandrov & K. Anokhin (Eds.), *Seventh International Conference on Cognitive Science: Abstracts. Svetlogorsk, June 20-24 2016. (pp. 93-95)*. Institut Psikhologii RAN.

Akinina, Yu., Iskra, E., Ivanova, M., Grabovskaya, M., Isaev, D., Korkina, I., Malyutina, S., Sergeeva, N. (2014). *Biblioteka stimulov «Sushhestvitel'noe i ob"ekt»: normirovanie psiholingvisticheskih parametrov* [Stimulus database 'Noun and object': normative data collection for psycholinguistic variables]. In A. Kibrik et al. (Eds.), *Sixth International Conference on Cognitive Science: Abstracts. Kaliningrad, June 23-27 2014. Issue 6* (pp. 112-114).

Armstrong, E. (2000). Aphasic discourse analysis: The story so far. *Aphasiology, 14*(9), 875-892.

Bailyn, J. F. (Ed.). (2011). A descriptive overview of Russian word order. In *The Syntax of Russian* (pp. 237–291). https://doi.org/DOI: 10.1017/CBO9780511984686.008

Brookshire, R. H., & Nicholas, L. E. (1993). *The Discourse Comprehension Test.* Tucson, AZ: Communication Skill Builders, A Division of The Psychological Corporation.

Bryant, L., Ferguson, A., & Spencer, E. (2016). Linguistic analysis of discourse in aphasia: A review of the literature. *Clinical Linguistics & Phonetics*, *30*(7), 489-518.

Caplan, D., & Evans, K. L. (1990). The effects of syntactic structure on discourse comprehension in patients with parsing impairments. *Brain and Language*, *39*(2), 206–234.

Caramazza, A., & Berndt, R. S. (1978). Semantic and syntactic processes in aphasia: a review of the literature. *Psychological Bulletin, 85*(4), 898.

Carpenter, P. (1995). Language Comprehension: Sentence and Discourse Processing. *Annual Review of Psychology*, *46*(1), 91–120.

Cho-Reyes, S., & Thompson, C. K. (2012). Verb and sentence production and comprehension in aphasia: Northwestern Assessment of Verbs and Sentences (NAVS). *Aphasiology, 26*(10), 1250-1277.

Coady, J. A., & Evans, J. L. (2008). Uses and interpretations of non-word repetition tasks in children with and without specific language impairments (SLI). *International Journal of Language & Communication Disorders, 43*(1), 1-40.

Deger, K., & Ziegler, W. (2002). Speech motor programming in apraxia of speech. *Journal of phonetics, 30*(3), 321-335.

De Renzi, E., & Flaglioni, P. (1978). Normative data and screening power of a shortened version of the Token Test. *Cortex, 14*, 41-9.

Druks, J., & Masterson, J. (2000). *An Object and Action Naming Battery*. Hove: Psychology Press.

Dyakonova, M. (2009). *A phase-based approach to Russian free word order.* Doctoral dissertation. University of Amsterdam.

Estes, K. G., Evans, J. L., & Else-Quest, N. M. (2007). Differences in the nonword repetition performance of children with and without specific language impairment: A meta-analysis. *Journal of Speech, Language, and Hearing Research*, *50*, 177–195.

Ferstl, E. C., Walther, K., Guthke, T., & von Cramon, D. Y. (2005). Assessment of Story Comprehension Deficits After Brain Damage. *Journal of Clinical and Experimental Neuropsychology*, *27*(3), 367–384.

Gathercole, S. E., Willis, C. S., Baddeley, A. D., & Emslie, H. (1994). The children's test of nonword repetition: A test of phonological working memory. *Memory, 2*(2), 103-127.

Gathercole, S. E. (2006). Nonword repetition and word learning: The nature of the relationship. *Applied Psycholinguistics, 27*, 513- 543.

Goldinger, S. D. (1996). Auditory lexical decision. *Language and Cognitive Processes, 11*(6), 559-568.

Goodglass, H., Kaplan, E., & Barresi, B. (2001). *The Boston Diagnostic Aphasia Examination (BDAE), 3rd ed*. Baltimore: Lippincott Williamson & Wilkins.

Gow Jr, D. W., & Caplan, D. (1996). An examination of impaired acoustic–phonetic processing in aphasia. *Brain and Language, 52*(2), 386-407.

Haley, K. L., & Jacks, A. (2014). Single-word intelligibility testing in aphasia: Alternate forms reliability, phonetic complexity and word frequency. *Aphasiology, 28*(3), 320-337.

Huber, W., Poeck, K., Weniger, D., & Willmes, K. (1983). *AAT-Aachener aphasie test*. Hogrefe, Göttingen.

Kay, J., Lesser, R., & Coltheart, M. (2009). *PALPA: Psycholinguistic assessments of language processing in aphasia*. Hove: Psychology Press.

Kertesz, A. (1982). *Western Aphasia Battery*. New York: Grune and Stratton.

Kintsch, W., & Van Dijk, T. A. (1978). Toward a model of text comprehension and production. *Psychological Review*, *85*(5), 363–394.

Lyashevskaya, O. N., & Sharoff, S. (2009). *Frequency Dictionary of the Modern Russian Language.* Azbukovnik.

Mar, R. A. (2004). The neuropsychology of narrative: story comprehension, story production and their interrelation. *Neuropsychologia*, *42*(10), 1414–1434.

Mätzig, S., Druks, J., Masterson, J., & Vigliocco, G. (2009). Noun and verb differences in picture naming: Past studies and new evidence. *Cortex, 45*(6), 738-758.

Mulligan, N. W. (2001). Word frequency and memory : Effects on absolute versus relative order memory and on item memory versus order memory. *Memory & Cognition, 29*(7), 977–985.

Nicholas, L. E., & Brookshire, R. H. (1995). Comprehension of spoken narrative discourse by adults with aphasia, right-hemisphere brain damage, or traumatic brain injury. *American Journal of Speech-Language Pathology*, *4*(3), 69-81.

Nozari, N., Kittredge, A. K., Dell, G. S., & Schwartz, M. F. (2010). Naming and repetition in aphasia: Steps, routes, and frequency effects. *Journal of Memory and Language, 63*(4), 541-559.

Olness, G. S. (2006). Genre, verb, and coherence in picture-elicited discourse of adults with aphasia. *Aphasiology*, *20*(02-04), 175-187.

Prins, R., & Bastiaanse, R. (2004). Analyzing the spontaneous speech of aphasic speakers. *Aphasiology*, *18*(12), 1075–1091.

Pritchard, M., Hilari, K., Cocks, N., & Dipper, L. (2017). Reviewing the quality of discourse information measures in aphasia. *International Journal of Language & Communication Disorders, 52*(6), 689-732.

Saito, A., Yoshimura, T., Itakura, T., & Ralph, M. A. L. (2003). Demonstrating a wordlikeness effect on nonword repetition performance in a conduction aphasic patient. *Brain and Language, 85*(2), 222-230.

Schumacher, R., Cazzoli, D., Eggenberger, N., Preisig, B., Nef, T., Nyffeler, T., ... & Müri, R. M. (2015). Cue recognition and integration–eye tracking evidence of processing differences in sentence comprehension in aphasia. *PloS one, 10*(11), e0142853.

Shankweiler, D., & Harris, K. S. (1966). An experimental approach to the problem of articulation in aphasia. *Cortex, 2*(3), 277-292.

Soloukhina, O. A., & Ivanova, M. V. (2018). Investigating comprehension of nouns and verbs: is there a difference? *Aphasiology*, *32*(2), 183–203. https://doi.org/10.1080/02687038.2017.1396572

Swinburn, K., Porter, G., & Howard, D. (2004). *Comprehensive Aphasia Test*. Hove: Psychology Press.

Thompson, C. K. (2012). *Northwestern assessment of verbs and sentences (NAVS).* Evanston, IL: Northwestern University.

Trost, J. E., & Canter, G. J. (1974). Apraxia of speech in patients with Broca's aphasia: A study of phoneme production accuracy and error patterns. *Brain and Language, 1*(1), 63-79.

Tsvetkova, L. S., Akhutina, T. V., & Pylaeva, N. M. (1981). Metodika otsenki rechi pri afazii [Method of quantitative assessment of the speech symptoms in aphasiaology to evaluate speech in aphasia]. Moscow: Moskovskiy Gosudarstvennyiy Universitet.

Van Dijk, T. A., & Kintsch, W. (1983). *Strategies of Discourse Comprehension*.

Vigliocco G., Vinson D. P., Druks J., Barber H., & Cappa S. F. (2011). Nouns and verbs in the brain: A review of behavioural, electrophysiological, neuropsychological and imaging studies. *Neuroscience and Biobehavioral Reviews, 35*(3), 407 – 426. doi:10.1016/j.neubiorev.2010.04.007

Whitworth, A., Webster, J., & Howard, D. (2005). Identifying and characterizing impairments: principles and evidence. In A. Whitworth, J. Webster, & D. Howard (Eds.), *A cognitive neuropsychological approach to assessment and intervention in aphasia* (pp. 11–22). Hove, East Sussex: Taylor & Francis.

Wilson, S. M., Eriksson, D. K., Schneck, S. M., & Lucanie, J. M. (2018). A quick aphasia battery for efficient, reliable, and multidimensional assessment of language function. *PloS one, 13*(2), e0192773.

Yamadori, A., & Ikumura, G. (1975). Central or conduction aphasia in a Japanese patient. *Cortex, 11*, 73–82.
