# Supplementary material for: The Russian Aphasia Test: The first comprehensive, quantitative, standardized, and computerized aphasia language battery in Russian
Source: PLoS One. 2021 Nov 18;16(11):e0258946. doi: 10.1371/journal.pone.0258946 (PMC8601577; doi:10.1371/journal.pone.0258946)
Supplement: S1 Table — (PDF) [file pone.0258946.s002.pdf]

S2 Table. Descriptive statistics for accuracy subtest scores (% correct) of the RAT for the control group of neurologically healthy individuals (NHI) and the main group of people with aphasia (PWA) for each age cohort.

Subtest	Age	Group	N	Range	Mean (SD)	Median (IQR)
Nonword Discrimination	young	NHI	69	77.27 - 100	96.64 (4.45)	100 (4.55)
		PWA	43	0 - 100	81.08 (25.62)	90.91 (22.73)
	elderly	NHI	37	81.82 - 100	95.44 (4.8)	95.45 (4.55)
		PWA	40	0 - 100	72.84 (32.1)	90.91 (36.36)
Lexical Decision	young	NHI	69	91.67 - 100	99.4 (1.79)	100 (0)
		PWA	44	0 - 100	87.12 (22.5)	95.83 (12.5)
	elderly	NHI	36	83.33 - 100	97.69 (4.28)	100 (4.17)
		PWA	40	0 - 100	86.56 (21.88)	95.83 (8.33)
Noun Comprehension	young	NHI	69	91.67 - 100	99.82 (1.12)	100 (0)
		PWA	44	70.83 - 100	96.78 (6.35)	100 (4.17)
	elderly	NHI	37	91.67 - 100	99.1 (2)	100 (0)
		PWA	41	37.5 - 100	91.06 (13.84)	95.83 (12.5)
Verb Comprehension	young	NHI	69	83.33 - 100	98.07 (3.61)	100 (4.17)
		PWA	44	54.17 - 100	91.38 (11.83)	95.83 (12.5)
	elderly	NHI	37	87.5 - 100	97.97 (3.05)	100 (4.17)
		PWA	41	54.17 - 100	89.43 (12.36)	91.67 (12.5)
Sentence Comprehension	young	NHI	69	87.5 - 100	98.49 (2.47)	100 (4.17)
		PWA	44	45.83 - 100	81.63 (16.31)	87.5 (26.04)
	elderly	NHI	37	83.33 - 100	97.52 (4.1)	100 (4.17)
		PWA	40	12.5 - 100	78.85 (19.16)	79.17 (29.17)
Discourse Comprehension	young	NHI	69	62.5 - 100	94.36 (8.74)	100 (12.5)
		PWA	40	0 - 100	63.44 (31.82)	75 (37.5)
	elderly	NHI	37	50 - 100	86.15 (14.06)	87.5 (25)
		PWA	38	0 - 100	56.58 (32.33)	62.5 (50)
Nonword Repetition	young	NHI	68	89.58 - 100	96.62 (2.47)	97.92 (2.08)
		PWA	44	0 - 100	62.74 (35.41)	72.92 (69.27)
	elderly	NHI	37	81.25 - 100	93.43 (4.3)	93.75 (4.17)
		PWA	40	0 - 100	51.24 (36.26)	45.83 (63.95)
Word Repetition	young	NHI	69	95.65 - 100	99.6 (1.05)	100 (0)
		PWA	44	0 - 100	79.16 (31.29)	93.75 (28.13)
	elderly	NHI	36	95.83 - 100	99.59 (0.97)	100 (0)
		PWA	41	0 - 100	70.57 (37.07)	89.58 (39.58)

Subtest	Age	Group	N	Range	Mean (SD)	Median (IQR)
Sentence Repetition	young	NHI	69	80.95 - 100	97.15 (4.3)	98.41 (3.97)
		PWA	44	0 - 99.21	54.5 (37.86)	64.29 (74.4)
	elderly	NHI	37	81.75 - 100	94.92 (4.85)	96.03 (4.76)
		PWA	41	0 - 100	43.57 (37.67)	47.62 (75.4)
Object Naming	young	NHI	68	83.33 - 100	97.86 (3.71)	100 (4.17)
		PWA	44	0 - 100	68.42 (33.42)	87.5 (54.17)
	elderly	NHI	34	83.33 - 100	96.3 (5.51)	100 (4.45)
		PWA	40	0 - 100	61.25 (36.41)	79.17 (56.25)
Action Naming	young	NHI	69	87.5 - 100	98.25 (2.8)	100 (4.17)
		PWA	43	0 - 100	68.86 (34.94)	83.33 (48.91)
	elderly	NHI	36	70.83 - 100	96.27 (6.51)	100 (4.17)
		PWA	40	0 - 100	57.96 (34.54)	72.92 (55.21)
Sentence Production	young	NHI	66	78.13 - 100	94.51 (4.38)	95.83 (5.21)
		PWA	39	0 - 93.75	47.73 (33.47)	52.78 (57.29)
	elderly	NHI	28	55.21 - 100	87.67 (9.51)	89.58 (4.17)
		PWA	37	0 - 83.33	38.62 (31.74)	45.83 (67.71)
Discourse Production	young	NHI	69	75 - 100	90.65 (6.3)	90 (5)
		PWA	38	0 - 95	46.97 (27.67)	50 (33.75)
	elderly	NHI	35	75 - 100	91.14 (5.83)	90 (5)
		PWA	38	0 - 80	45 (30.78)	57.5 (70)

Note. *N* = number of participants used to calculate the values.
