# Supplementary material for: The Russian Aphasia Test: The first comprehensive, quantitative, standardized, and computerized aphasia language battery in Russian
Source: PLoS One. 2021 Nov 18;16(11):e0258946. doi: 10.1371/journal.pone.0258946 (PMC8601577; doi:10.1371/journal.pone.0258946)
Supplement: S2 Table — (PDF) [file pone.0258946.s003.pdf]

S3 Table. Cutoffs scores (%) and identified 34th and 67th percentiles for the PWA group used to determine aphasia severity ranks in each subtest and the General Aphasia Quotient (GAQ).

Subtest	Age	N	34th percentile	67th percentile	Cutoff
Nonword Discrimination	young	45	58.91	84	90.91
	elderly				86.36
Lexical Decision	young	50	86.08	91.67	95.83
	elderly				94.38
Noun Comprehension	young	40	88.58	95.83	95.83
	elderly				95.83
Verb Comprehension	young	49	83.33	91.67	95.83
	elderly				94.79
Sentence Comprehension	young	64	68.42	84.21	95.83
	elderly				91.67
Discourse Comprehension	young	49	37.5	62.5	75
	elderly				62.5
Nonword Repetition	young	61	23.75	66.67	91.67
	elderly				85
Word Repetition	young	56	60.42	89.58	97.66
	elderly				97.92
Sentence Repetition	young	67	9.08	61.02	90.48
	elderly				85.24
Object Naming	young	56	36.25	78.54	91.67
	elderly				83.33
Action Naming	young	63	33.67	79.17	93.13
	elderly				87.5
Sentence Production	young	67	19.79	60.42	87.71
	elderly				74.27
Discourse Production	young	73	45	61.2	80
	elderly				80
GAQ	young	61	61.17	83.45	92.95
	elderly				89.91

Note. *N* = number of participants used to calculate the severity ranks, equal to the combined number of young and elderly PWA with abnormal performance on a given subtest (i.e., performing at or below the cutoff score on each subtest). The 34th and the 67th percentiles are identical for both age cohorts. The three severity ranks were defined in the same way for the RAT subtest scores and the GAQ values, namely, Mild: 67th percentile ≤ Xi ≤ cutoff; Moderate: 34th percentile < Xi < 67th percentile; and Severe: 0 ≤ Xi ≤ 34th percentile, where Xi is the individual participant's accuracy score on a given subtest or the GAQ.
