# Supplementary material for: The Russian Aphasia Test: The first comprehensive, quantitative, standardized, and computerized aphasia language battery in Russian
Source: PLoS One. 2021 Nov 18;16(11):e0258946. doi: 10.1371/journal.pone.0258946 (PMC8601577; doi:10.1371/journal.pone.0258946)
Supplement: S3 Table — (PDF) [file pone.0258946.s004.pdf]

S4 Table. Item passing rates (item difficulty) of the RAT subtests based on the data of the PWA group.

item # / statistic	Nonword Discrimination	Lexical Decision	Noun Comprehen-sion	Verb Comprehen-sion	Sentence Comprehen-sion	Discourse Comprehen-sion	Nonword Repetition	Word Repetition	Sentence Repetition	Object Naming	Action Naming	Sentence Production
1	0.82	0.89	0.89	0.96	0.68	0.78	0.51	0.85	0.68	0.73	0.81	0.37
2	0.64	0.89	0.94	1	0.87	0.63	0.62	0.82	0.74	0.71	0.68	0.55
3	0.75	0.88	0.93	0.93	0.77	0.65	0.58	0.87	0.67	0.74	0.84	0.58
4	0.77	0.89	0.95	0.89	0.95	0.49	0.67	0.8	0.54	0.81	0.7	0.46
5	0.8	0.92	0.96	0.92	0.83	0.71	0.57	0.78	0.52	0.77	0.62	0.5
6	0.83	0.86	0.95	0.87	0.82	0.38	0.61	0.83	0.52	0.64	0.61	0.68
7	0.84	0.94	0.91	0.88	0.77	0.45	0.57	0.83	0.5	0.73	0.78	0.52
8	0.81	0.94	0.94	0.71	0.86	0.72	0.71	0.76	0.57	0.62	0.69	0.49
9	0.83	0.9	0.96	0.92	0.94	-	0.58	0.78	0.54	0.67	0.54	0.41
10	0.83	0.79	0.92	0.94	0.9	-	0.57	0.78	0.3	0.74	0.67	0.51
11	0.78	0.85	0.96	0.89	0.76	-	0.76	0.73	0.3	0.58	0.7	0.44
12	0.77	0.93	0.87	0.84	0.68	-	0.61	0.81	0.29	0.67	0.64	0.44
13	0.53	0.9	0.98	0.98	0.95	-	0.62	0.79	-	0.7	0.63	0.38
14	0.84	0.85	0.99	0.95	0.69	-	0.54	0.8	-	0.63	0.57	0.42
15	0.55	0.56	0.95	0.93	0.88	-	0.65	0.73	-	0.65	0.64	0.36
16	0.81	0.9	0.98	0.8	0.58	-	0.58	0.7	-	0.6	0.63	0.38
17	0.83	0.92	0.96	0.96	0.8	-	0.62	0.66	-	0.47	0.71	0.46
18	0.77	0.95	0.93	0.93	0.99	-	0.46	0.72	-	0.62	0.6	0.37
19	0.75	0.9	0.98	0.98	0.62	-	0.54	0.7	-	0.62	0.63	0.43
20	0.77	0.81	0.94	0.78	0.71	-	0.44	0.71	-	0.45	0.54	0.35
21	0.84	0.87	0.89	0.91	0.92	-	0.45	0.63	-	0.63	0.7	0.34
22	0.8	0.87	0.89	0.92	0.65	-	0.55	0.66	-	0.74	0.49	0.41
23	-	0.86	0.92	0.94	0.77	-	0.51	0.51	-	0.54	0.45	0.44
24	-	0.77	0.95	0.88	0.86	-	0.4	0.65	-	0.54	0.43	0.37

item # / statistic	Nonword Discrimination	Lexical Decision	Noun Comprehen-sion	Verb Comprehen-sion	Sentence Comprehen-sion	Discourse Comprehen-sion	Nonword Repetition	Word Repetition	Sentence Repetition	Object Naming	Action Naming	Sentence Production
Mean	0.77	0.87	0.94	0.90	0.80	0.60	0.57	0.75	0.51	0.65	0.64	0.44
SD	0.09	0.08	0.03	0.07	0.11	0.14	0.08	0.08	0.15	0.09	0.10	0.08
Min	0.53	0.56	0.87	0.71	0.58	0.38	0.4	0.51	0.29	0.45	0.43	0.34
Max	0.84	0.95	0.99	1	0.99	0.78	0.76	0.87	0.74	0.81	0.84	0.68

Note. Item difficulty was not computed for Discourse Production as it only has one item.
